# Coupling Long‐Range Facet Junction and Interfacial Heterojunction via Edge‐Selective Deposition for High‐Performance Z‐Scheme Photocatalyst

**DOI:** 10.1002/advs.202200346

**Published:** 2022-04-24

**Authors:** Xuan Li, Shoaib Anwer, Qiangshun Guan, Dalaver H. Anjum, Giovanni Palmisano, Lianxi Zheng

**Affiliations:** ^1^ Department of Mechanical Engineering Khalifa University of Science and Technology Abu Dhabi 127788 United Arab Emirates; ^2^ Research and Innovation on CO2 and H2 (RICH) Center Khalifa University of Science and Technology Abu Dhabi 127788 United Arab Emirates; ^3^ Department of Physics Khalifa University of Science and Technology Abu Dhabi 127788 United Arab Emirates; ^4^ Department of Chemical Engineering Khalifa University of Science and Technology Abu Dhabi 127788 United Arab Emirates

**Keywords:** 2D material, facet junction, heterojunction, photocatalyst, photodegradation, selective growth, Z‐scheme

## Abstract

The construction of photocatalytic systems that have strong redox capability, effective charge separation, and large reactive surfaces is of great scientific and practical interest. Herein, an edge‐connected 2D/2D Z‐scheme system that combines the facet junction and the interfacial heterojunction to achieve effective long‐range charge separation and large reactive surface exposure is designed and fabricated. The heterostructure is realized by the selective growth of 2D‐layered MoS_2_ nanoflakes on the edge‐sites of thin TiO_2_ nanosheets via an Au‐promoted photodeposition method. Attributed to the synergetic coupling of the facet junction and the interfacial heterojunction that assures the effective charge separation, and the tremendous but physically separated reactive sites offered by layered MoS_2_ and highly‐exposed (001) facets of TiO_2_, respectively, the artificial Z‐scheme exhibits excellent photocatalytic performance in photodegradation tests. Moreover, the junctional plasmonic Au nanoclusters not only act as electron traps to promote the edge‐selective synthesis but also generate “hot electrons” to further boost photocatalytic performance. The Z‐scheme charge‐flow direction in the heterostructure and the roles of electrons and holes are comprehensively studied using in situ irradiated X‐ray photoelectron spectroscopy and photodegradation tests. This work offers a new insight into designing high‐performance Z‐scheme photocatalytic systems.

## Graphical abstract

An efficient MoS_2_/Au/TiO_2_ Z‐scheme photocatalytic system is fabricated by Au‐induced selective growth of MoS_2_ nanoflakes on the edge‐sites of TiO_2_ nanosheets. The ternary structure synergistically couples the facet junction and the heterojunction to separate the photogenerated charges in relays and maintains the highly exposed surfaces for redox reactions. The junctional Au nanoclusters generate “hot electrons” to further boost photocatalytic performance.

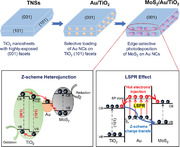

## 1 Introduction

Two of the biggest challenges confronting the world today are the global crises of clean energy supply and environmental pollution. To address these concerns, one of the most promising and sustainable technologies is sunlight‐assisted semiconductor‐based photocatalysis, which uses free solar energy to produce clean energy and for the removal of environmental pollutants.^[^ ^1^ ^]^ An ideal semiconductor photocatalyst should have strong redox capability, effective charge separation, and a large reactive surface area.^[^ ^2^ ^]^ To boost the redox capability, constructing Z‐scheme heterojunction has proven to be the most effective strategy because it could take the synergistic effects of two semiconducting components to keep the high‐energy holes/electrons for redox reactions.^[^ ^3^ ^]^ The effective charge separation could be realized by facet junctions^[^ ^4^ ^]^ or heterojunctions,^[^ ^5^ ^]^ with facet junctions having weak but relatively long‐range charge separation while heterojunctions possessing strong but local charge separation. The large reactive surface area is commonly achieved by employing low‐dimensional, especially 2D nanomaterials.^[^ ^6^ ^]^ Irrespective of the progress made in each of these areas, it remains an extreme challenge to construct a 2D material‐based Z‐scheme photocatalytic system with a proper geometry so that all of the three merits exist in one unique catalytic system.

Interfacial design and selective synthesis are two major obstacles toward this goal. To obtain a Z‐scheme heterojunction for high redox capability, two constituent semiconductors must have well‐matched band alignment, intimate contact, and a dedicated charge transfer channel.^[^ ^2^ ^]^ However, interfacial defects, functional groups, adsorbents, etc., could easily alter these interfacial properties, resulting in the formation of a Type II rather than a Z‐scheme heterojunction.^[^ ^7^ ^]^ In order to alleviate this uncertainty, minor differences between surface sites or crystal facets have been utilized for selectively constructing Z‐scheme heterostructures. It was found that some sites of a material surface or some crystal facets of a semiconductor were more favorable for the accumulation of electrons while some were prone to act as hole traps.^[^ ^4^ ^]^ This difference could be an intrinsic feature of the materials or induced by surface modification via ion or particle decoration. For example, CdS was selectively deposited at the electron transfer sites of g‐C_3_N_4_ to regulate the electron flow direction.^[^ ^8^ ^]^ Selective growth of CdS on (001) or (101) TiO_2_ facets was controlled by attaching/detaching F^−^ ions.^[^ ^9^ ^]^ Core–shell heterojunctions were achieved by Au nanoparticle‐mediated growth.^[^ ^10^ ^]^ TiO_2_ hollow nanoboxes were utilized to force (101) facets to be exposed to the outmost of the cluster structures, forming TiO_2_(101)/g‐C_3_N_4_ contacted heterostructures.^[^ ^11^ ^]^ Cross‐stacked TiO_2_ nanostructures were used to expose (101) facets for fast and easy access to the precursors during photodeposition (PD) and to avoid the stacking growth of MoS_2_ on (001) facets.^[^ ^12^ ^]^ These strategies demonstrate great success in constructing different types of Z‐scheme heterojunctions, including randomly deposited, site‐selectively deposited, and facet‐selectively deposited Z‐schemes, but still fall short of obtaining high‐performance photocatalysts with both efficient charge separation and large reactive surface area. More specifically, in randomly or site‐selectively deposited Z‐scheme structures, the second semiconductor generally covers the surface of the first semiconductor in the format of nanoparticles or nanoclusters (NCs), so the increase of surface area for reduction (or oxidation) is at the expense of the decrease of the surface area for oxidation (or reduction). Furthermore, the spatial distance between these NCs is extremely short, which increases the charge recombination via environmental routes (e.g., backward redox in the liquid phase). Although the heterojunction itself has effective charge separation capability, the external recombination routes will compromise the overall charge separation efficiency. Consequently, such photocatalysts usually need sacrificial agents to guarantee their photocatalytic performance. In facet‐selectively deposited Z‐scheme structures, a complex morphology of the first semiconductor, which involves a complex fabrication process, is required to confine the access of precursors of the second semiconductor during the selective synthesis/construction. This confining mechanism, as well as the complex geometry, will also limit the access to the target molecules in photocatalytic applications, resulting in a significant decrease in the active surface area of the catalyst and thus the extent of its utilization.

Herein, we design a 2D material‐based Z‐scheme photocatalyst that constructively couples the facet junction and heterojunction to simultaneously achieve high redox capability, efficient charge separation, and large reactive surface area. 2D anatase TiO_2_ nanosheets (TNSs) with highly‐exposed (001) facets and layered MoS_2_ nanoflakes (NFs) are employed as two‐component materials. Au NCs are first decorated on the edge surfaces (i.e., (101) facets) of TNSs via a diffusion and spatial confinement strategy. MoS_2_ is then selectively deposited on the Au NCs and TiO_2_ edge surfaces via a simple PD method. The resultant structure not only takes advantages of the combinative effect from the facet junction and heterojunction to maximize the charge separation and form a Z‐scheme structure but also physically separates the redox reactive surfaces, leaving the highly‐exposed (001) facets of 2D TNSs for oxidation and MoS_2_ for reduction reactions. While the Au NCs implanted between two semiconductors not only serve as a mediator to facilitate the fast migration of photogenerated electron holes at the heterointerface to ensure an effective Z‐scheme charge transfer but also act as a source of “hot electron” injection to improve the photocatalytic performance. Such a photocatalyst system is extremely beneficial for photodegradation applications that have diverse and unknown pollutants.

## 2 Results and Discussion

### 2.1 Design and Edge‐Selective Construction of MoS_2_/Au/TiO_2_ Heterogeneous Photocatalyst

**Figure 1:**
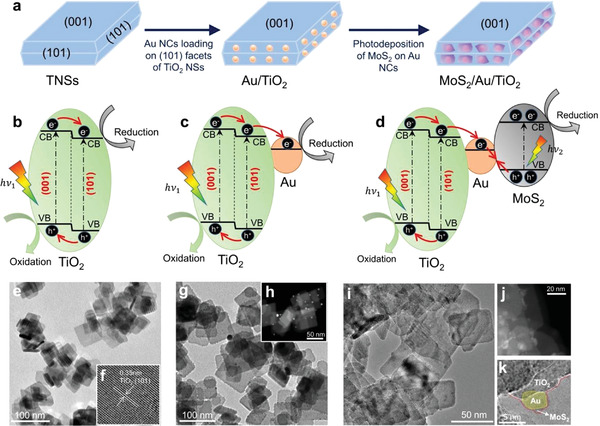
a) Stepwise schematic diagram of design concept and synthesis process of MoS_2_/Au/TiO_2_ heterostructures. The energy band diagram and corresponding charge transfer route for b) TNSs, c) Au/TiO_2_, and d) MoS_2_/Au/TiO_2_. e) TEM image of TNSs, inset f) HRTEM image of TNSs showing the lattice spacing. g) Bright‐field (BF) HRTEM image and h) high‐angle annular dark‐field scanning TEM (HAADF‐STEM) image of edge‐site deposited Au/TiO_2_; i) TEM, j) HAADF‐STEM, and k) BF‐HRTEM images of selectively deposited MoS_2_/Au/TiO_2_ nanostructures.

The idea of junction coupling was realized by constructing an edge‐connected MoS_2_/Au/TiO_2_ (MS/Au/T) heterogeneous photocatalyst. The schematic design and stepwise fabrication process are illustrated in **Figure** **1**a. In the first step, rectangular‐shaped anatase TNSs with highly‐exposed (001) facets were prepared via a hydrothermal process.^[^ ^13^ ^]^ In the second step, Au atoms and fine nanoparticles were dispersed on the TNSs via solution phase synthesis. The dispersed Au particles were then turned into selectively deposited Au NCs on the edge‐sites, that is, (101) facets of TNSs after controlled calcination treatment to obtain Au/TiO_2_ (Au/T) heterostructures. Mechanistically, Au ions were first reduced into Au atoms or small particles that were randomly distributed over the TNSs surfaces and then transformed to NCs and selectively accumulated on (101) facets via diffusion and spatial confinement mechanism.^[^ ^14^ ^]^ In the last step, MoS_2_ was selectively deposited via a modified PD method at room temperature.^[^ ^12^ ^]^ Au/T was initially mixed with an aqueous solution containing MoS_4_^2−^ and then irradiated with simulated solar light. Interestingly, it was found that MoS_2_ preferably grew on Au NCs first, then on (101) facets of TNSs to form ternary MS/Au/T heterostructures. Notably, Au played a critical role in the PD process, because it offered strong affinity with S,^[^ ^10^ ^]^ trapped electrons from TiO_2_ (101) facets, and produced more electrons via localized surface plasmon resonance (LSPR). Therefore, it accumulated more electrons to amplify the potential difference between (001) and (101) facets of TNSs, and thus promoted the selective PD of MoS_2_ on Au NCs and the edges of TNSs.

To illustrate the charge carrier's migration principle, the energy band diagrams of TNSs, Au/T, and MS/Au/T are shown in Figure 1b–d. Under light irradiation, the photogenerated electrons and holes transfer to (101) and (001) facets of TNSs respectively, forming a facet junction (Figure 1b).^[^ ^15^ ^]^ Since the work function of Au is larger than that of TiO_2_,^[^ ^3^ ^]^ the accumulated photogenerated electrons at the interface transfer from TiO_2_ (101) facets to Au NCs, establishing a consecutive electron flow path: TiO_2_ (001) facet → TiO_2_ (101) facet → Au NCs (Figure 1c), which facilitates an efficient charge separation in Au/T heterostructure. In MS/Au/T, Au NCs act as the mediator to recombine the photoinduced electrons from the conduction band (CB) of TiO_2_ (101) facets with the holes from the valence band (VB) of MoS_2_, forming a Z‐scheme charge transfer that leaves the electrons in CB of MoS_2_ and holes in VB of TiO_2_ (001) facets with higher energies for redox reactions (Figure 1d).^[^ ^10^ ^]^ This Z‐scheme boosts the charge separation to have a higher photocatalytic efficiency and can be applied in various catalytic reactions with high redox potentials, owing to its well‐retained strong redox ability. Particularly, in our system, the photoinduced holes mainly accumulate on the TiO_2_ (001) facets with a large area, which offer highly‐exposed oxidation sites and thus promote the photocatalytic reaction. In addition, the spatially separated reduction (MoS_2_) and oxidation (TiO_2_ [001] facets) active sites allow the photocatalyst system to participate in reduction and oxidation reactions simultaneously, beneficial for the photodegradation applications that have diverse and unknown pollutants.

The morphologies of TNSs, Au/T, and MS/Au/T were examined by scanning electron microscopy (SEM) and high‐resolution transmission electron microscopy (HRTEM) analysis. As depicted in Figure 1e and Figure S1, Supporting Information, TNSs exhibited a 2D rectangular shape with a mean length of ≈60 nm and thickness of ≈6 nm. Indexed lattice spacing of 0.35 nm (see inset Figure 1f) from TNS surfaces and 0.235 nm (Figure S1b, Supporting Information) from the vertical cross‐sectional image of TNSs were identified and correspond to the (101) and (001) planes of the crystalline anatase phase of TiO_2_,^[^ ^13^, ^16^ ^]^ respectively, confirming that the synthesized TNSs have highly‐exposed (001) facets and thin (101) facets. Figure 1g,h and Figure S2a,b, Supporting Information, demonstrated that highly crystallized Au NCs were successfully deposited on the edge sites of TNSs with intimate contact. The observed lattice spacing of 0.235 nm (Figure S2c, Supporting Information) matched well with the (111) lattice plane of face‐centered cubic (FCC) Au.^[^ ^17^ ^]^ The transmission electron microscopy (TEM) image in Figure 1i and the high‐angle annular dark‐field scanning TEM (HADDF‐STEM) image in Figure 1j clearly displayed that MoS_2_ NFs were intimately deposited in a layered manner via photo‐assisted nucleation and growth process at room temperature. Red‐dotted line in Figure 1k disclosed that MoS_2_ NFs were thin and immature crystalline structures, but they formed the shell to cover the Au NCs (yellow circle) pre‐deposited on TiO_2_ (101) facets. A clearer HRTEM image showing two distinct interfaces of TiO_2_/Au and Au/MoS_2_ in MS/Au/T can be found in Figure S2d, Supporting Information.

The composition and crystal structure of the prepared photocatalysts were further investigated by powder X‐ray diffraction (XRD) and Raman spectroscopy. The obtained patterns of TNSs, Au/T, and MS/Au/T are shown in Figure S3, Supporting Information. As shown in Figure S3a, Supporting Information, the XRD diffraction peaks at 25.1°, 38.1°, 47.9°, and 75° were obvious in the as‐prepared three samples and could be respectively assigned to the (101), (004), (200), and (215) crystallographic planes of anatase TiO_2_, which well matched the reference pattern (JCPDS 21–1272).^[^ ^13^, ^16^ ^]^ Anatase TNSs were well‐retained without any phase change even after the deposition of Au NCs and MoS_2_ NFs. The diffraction peaks observed at 38.1°, 44.3°, 64.4°, and 77.5° in Au/T photocatalyst could be attributed to FCC Au (JCPDS 04–0784). The distinct (004) diffraction peak of TNSs indicates the dominant crystal growth along the (001) direction, which is typical for anatase TiO_2_ with highly‐exposed (001) facets.^[^ ^16^ ^]^ However, no obvious signals of MoS_2_ were observed in the XRD pattern of the MS/Au/T photocatalyst. This could be due to its amorphous‐like structure or poor crystallinity and thin‐layered 2D morphology or high dispersion on very small but crystalline Au NCs.^[^ ^18^ ^]^ The Raman studies further demonstrated this conjecture. As shown in Figure S3b, Supporting Information, the peaks at 143, 392, 511, and 632 cm^−1^ could be respectively assigned to *E*_g_, *B*_1g_, *A*_1g_, and *E*_g_ anatase tetragonal vibration modes of TiO_2_.^[^ ^19^ ^]^ The observed shift at 153 cm^−1^ corresponding to the *E*_g_ Raman mode of Au/T confirmed a strong interfacial contact and electronic interaction between TiO_2_ and Au.^[^ ^19^ ^]^ However, no characteristic peaks belonging to *E* ^1^_2g_ and *A*_1g_ of 2H‐MoS_2_ were found in the MS/Au/T spectrum, which confirmed the amorphous‐like structure and poor crystallinity of MoS_2_.^[^ ^20^ ^]^ As experimental evidence, the presence of MoS_2_ was proved by X‐ray photoelectron spectroscopy (XPS). The XPS spectrum of MS/Au/T exhibited two characteristic peaks at 229.04 and 232.14 eV (Figure S4, Supporting Information), attributed to the binding energies (BEs) of Mo 3*d*_5/2_ and Mo 3*d*_3/2_ for Mo^4+^ species.^[^ ^21^ ^]^ The BEs of Mo^4+^ 3*d* doublet demonstrated that MoS_2_ was a semiconducting 2H phase.^[^ ^22^ ^]^ The S 2*p*_3/2_ and S 2*p*_1/2_ doublets, respectively at 161.88 and 163.08 eV, were assigned to S^2−^ ions.^[^ ^21^ ^]^ These XPS results confirmed the presence of 2H‐MoS_2_ and supported the HRTEM investigations. Besides this, a contribution of Mo^5+^, Mo^6+^, and S_2_^2−^ was also evident^[^ ^21^ ^]^ and summarized in Table S1, Supporting Information.

The chemical states of the key elements and the formation of heterojunctions were further investigated by XPS. As shown in Figure S5, Supporting Information, the BEs of Ti 2*p* displayed obvious differences among TNSs, Au/T, and MS/Au/T. For Au/T, the BEs of Ti 2*p*_3/2_ and Ti 2*p*_1/2_ were respectively 458.47 and 464.17 eV, with splitting energy of 5.7 eV, manifesting the presence of tetragonal Ti^4+^,^[^ ^23^ ^]^ but both peaks were 0.11 eV lower than those of TNSs. This negative shift suggests strong interfacial interaction between Au and TiO_2_.^[^ ^24^ ^]^ After depositing MoS_2_ on Au/T, the BEs of Ti 2*p* showed an obvious positive shift (0.35 eV) compared to those of Au/T, which also suggests the strong interfacial interaction and indicates electrons moving out from TiO_2_ (into Au NCs). The XPS spectra of O 1*s* in TNSs, Au/T, and MS/Au/T can all be de‐convoluted into one major and two minor peaks (Figure S6, Supporting Information), assigned to the lattice oxygen (*O*_L_), surface oxygen vacancy (*O*_v_) and absorbed water molecules (*O*_w_), respectively.^[^ ^25^ ^]^ The O_L_ peak in Au/T exhibited a negative and positive shift compared to that in TNSs and MS/Au/T, respectively, which is consistent with the analysis of Ti 2*p* spectrum as described above. According to the XPS peak area, the content of *O*_v_ in the proportion of three oxygen peaks in TNSs, Au/T, and MS/Au/T was 3.5%, 6%, and 10.1% (Table S2, Supporting Information), respectively, demonstrating that more oxygen vacancies were formed in Au/T and especially in MS/Au/T. Two isolated peaks in the spectrum of Au 4*f* in Au/T, centered at 83.0 (Au 4*f*_7/2_) and 86.6 eV (Au 4*f*_5/2_) (Figure S7, Supporting Information), confirmed the existence of Au^0^ in the prepared photocatalysts.^[^ ^26^ ^]^ Positive shifts were observed in the peaks of Au 4*f* for MS/Au/T compared to those for Au/T, owing to the transfer of free electrons to recombine with the holes from MoS_2_.^[^ ^27^ ^]^ The above results demonstrate the existence of a strong interfacial interaction and the Z‐scheme heterojunction formed between MoS_2_, Au, and TiO_2_ in MS/Au/T photocatalyst, which are favorable for the charge carrier transport in photocatalysis.

### 2.2 Nucleation and Growth Mechanisms of MoS_2_ NFs on Au/T

**Figure 2:**
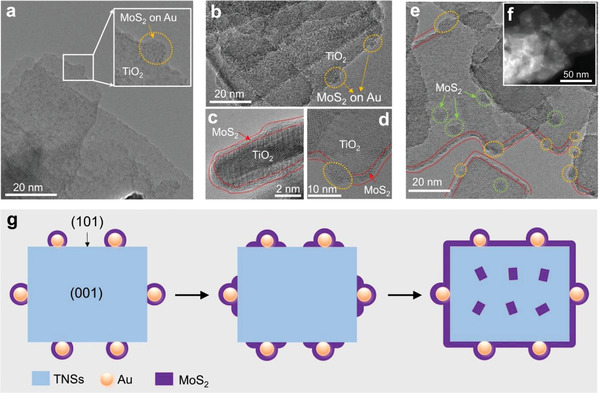
a) Nucleation and growth on Au NCs; b–d) extended growth on (101) facets of TNSs; e,f) excess growth on (001) facets of TNSs. g) Schematic illustration of the site‐selective nucleation, facet‐selective extension, and random excess growth process of MoS2 on Au/T heterostructure.

Detailed morphological studies showed that the deposition of MoS_2_ on Au/T could be divided into three stages: i) MoS_2_ primarily nucleated on the Au NCs and grew up into small‐size NFs, shown as small bumps or shells in **Figure** **2**a (marked in the yellow dotted circle); ii) then these MoS_2_ NFs expanded their growth to cover the (101) facets of TNSs, as shown in Figure 2b–d (red dotted line); iii) once (101) facets were fully covered, the excess MoS_2_ turned to deposit on the (001) facets of TNSs, as clearly shown in the BF‐HRTEM (green dotted circle in Figure 2e) and HAADF‐STEM (Figure 2f) images. This site‐selective nucleation, facet‐selective extension, and random excess growth process are schematically illustrated in Figure 2g.

The mechanism of nucleation and growth of MoS_2_ NFs can tentatively be explained as follows: under UV–vis/solar light irradiation, the photogenerated holes and electrons within TNSs were separated under the internal electric field formed between the (001) and (101) facets,^[^ ^15^ ^]^ resulted in the accumulation of electrons on (101) facets. Due to the intimate contact between Au NCs and TNSs, as well as the lower Fermi level of Au compared to TiO_2_,^[^ ^28^ ^]^ the electrons preferred to gather on the Au NCs (Figure 1c). In addition, MoS_2_ was formed from the photoreduction of MoS_4_^2−^ assisted by photo‐electrons,^[^ ^29^ ^]^ therefore, it is easy for MoS_2_ to nucleate and grow on the Au NCs that own enriched electrons and have a strong affinity to S. However, due to the small size of Au NCs, the growth of MoS_2_ was subsequently limited. MoS_2_ extended the growth in the other electron‐rich areas, that is, (101) facets of TNSs. Because of this extended growth pattern, a direct Z‐scheme charge transfer (as illustrated in Figure S8a, Supporting Information), in addition to the aforementioned Z‐scheme, will also form between TiO_2_ (101) facets and MoS_2_ due to their intimate contact. However, due to the strong electron trapping capability of Au NCs, TiO_2_ → Au → MoS_2_ Z‐scheme pattern still dominates the charge transfer process. After the surface of (101) facets were fully covered (only ≈6 nm wide), further growth of MoS_2_ could only occur on the (001) facets. In short, the selectivity of the growth is based on the electron density and the availability of the exposed surface area. Experimentally, it is interesting to note that the low initial concentration of MoS_4_^2−^ and shorter PD time are more favorable for selective and controlled growth.

### 2.3 Optimization of MoS_2_ in MS/Au/T System

In order to preserve the junction‐coupled Z‐scheme structure designed in Figure 1d, the aforementioned excess growth of MoS_2_ on (001) facets should be minimized because it decreases the oxidation surface area and forms a Type II structure (Figure S8b, Supporting Information), which weakens the redox capability. Although Au NCs greatly amplified the difference between (101) and (001) facets, selective growth and random growth are still competitive processes and the preference could be altered by other growth dynamics and reaction kinetics. A further in‐depth study on the growth rate and time is highly needed to optimize the MS/Au/T heterostructures for high photocatalytic performance.

**Figure 3:**
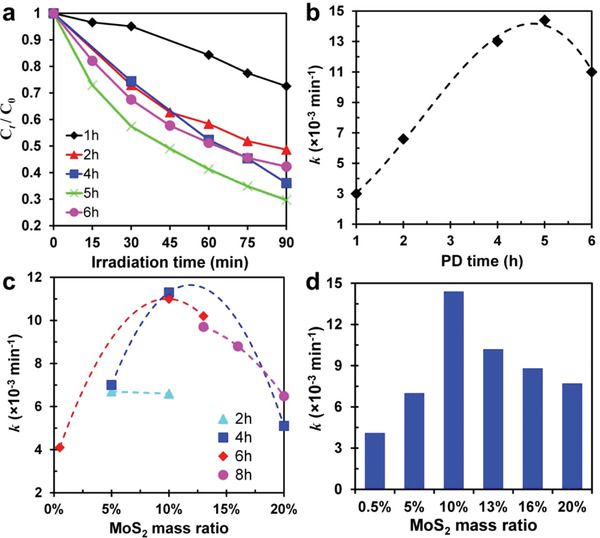
a) Photocatalytic activity and b) corresponding pseudo‐first‐order constants (*k*) of 10% MS/Au/T catalysts synthesized under different PD times: 1, 2, 4, 5, and 6 h. c) Comparison of the rate constants of prepared MS/Au/T catalysts at different mass ratios of MoS_2_ deposited under the same PD time; d) optimal rate constants of MS/Au/T catalysts with different MoS_2_ mass ratios.

Photocatalytic MB degradation experiments were then performed for the optimization of MS/Au/T photocatalysts under UV light illumination (360–370 nm) for 90 min. Catalyst samples were prepared at a fixed initial concentration of MoS_4_^2−^ but with different PD times. For clear guidance on the MoS_2_ amount and growth rate, the set of samples was named according to the theoretical mass ratio of MoS_2_ in the prepared sample, denoted as (%) MS/Au/T. For example, 10% MS/Au/T represents the set of samples synthesized at a fixed MoS_4_^2−^ concentration that yields 10% MoS_2_ if all MoS_4_^2−^ are converted into MoS_2_. **Figure** **3**a displayed the time profiles of *C_t_*/*C_0_* for 10% MS/Au/T catalysts with PD = 1, 2, 4, 5, and 6 h, respectively, where *C*_t_ is the MB concentration at irradiation time *t*, and *C*_0_ is the concentration at absorption equilibrium of the photocatalyst samples before irradiation. With the increase of PD time, the degradation rate increased first, until reaching the maximum value of 70.3% at PD = 5 h, and then decreased. The corresponding pseudo‐first‐order constant *k* (as calculated in Supporting Information) is shown in Figure 3b. This trend could be explained by the aforementioned nucleation and growth mechanism: For the samples with short PD time, their photocatalytic performance depended on the amount of deposited MoS_2_, which increased with PD time. As the PD time approached to ≈5 h, MoS_2_ fully covered the Au NCs and TiO_2_ (101) facets to form the proposed Z‐scheme that exhibited the best photocatalytic performance. However, as the PD time further increased, MoS_2_ started to grow on TiO_2_ (001) facets and disturbed the Z‐scheme charge transfer, leading to a decreased photocatalytic efficiency.

In addition to the PD time, the initial concentration of MoS_4_^2−^ also had a large impact on the growth of MoS_2_ and thus affected the photocatalytic efficiency. As shown in Figure 3c, we compared the rate constant *k* of MS/Au/T samples with different mass ratios of MoS_2_, which were synthesized at different initial concentrations of MoS_4_^2−^ but for the same PD time. For PD = 4, 6, and 8 h, when MoS_2_ was less than 10%, *k* increased with the increase of the MoS_2_ mass ratio. However, as MoS_2_ was greater than 10%, *k* decreased with the increase of MoS_2_. This observation could be explained as: when MoS_2_ was less than 10%, the photocatalytic efficiency was limited by the amount of MoS_2_ due to the insufficient growth of MoS_2_ on Au NCs and active TiO_2_ (101) facets. On the other hand, when MoS_2_ was larger than 10%, it was much easier and faster to grow on TiO_2_ (001) facets at a higher initial concentration of MoS_4_^2−^, which changed the charge transfer mechanism from Z‐scheme to Type II (formed between MoS_2_ and TiO_2_ [001] facets, as shown in Figure S8b, Supporting Information). In Type II charge transfer mechanism, the photogenerated electrons gathered on CB of TiO_2_ (101) facets and holes on VB of MoS_2_, respectively, both characterized by lower redox energy. Moreover, the excess growth of MoS_2_ on TiO_2_ (001) facets covered the active sites of holes for the catalytic reaction, further resulting in a low photocatalytic efficiency. However, at PD = 2 h, the values of *k* for 5% and 10% were almost the same, indicating that the growth of MoS_2_ was not enough, as explained before. After examining the overall trend of these curves, we have determined that 10% MoS_2_ is the optimum value in our designed experiment. This value could be affected by the morphology or exposed area ratio between (101) and (001) facets. The optimal *k* values for different mass ratios of MoS_2_ were summarized and shown in Figure 3d. Sample 10% MS/Au/T at PD = 5 h exhibited the best *k* (0.0144 min^−1^).

### 2.4 Confirmation of Z‐Scheme Charge Transfer in MS/Au/T

**Figure 4:**
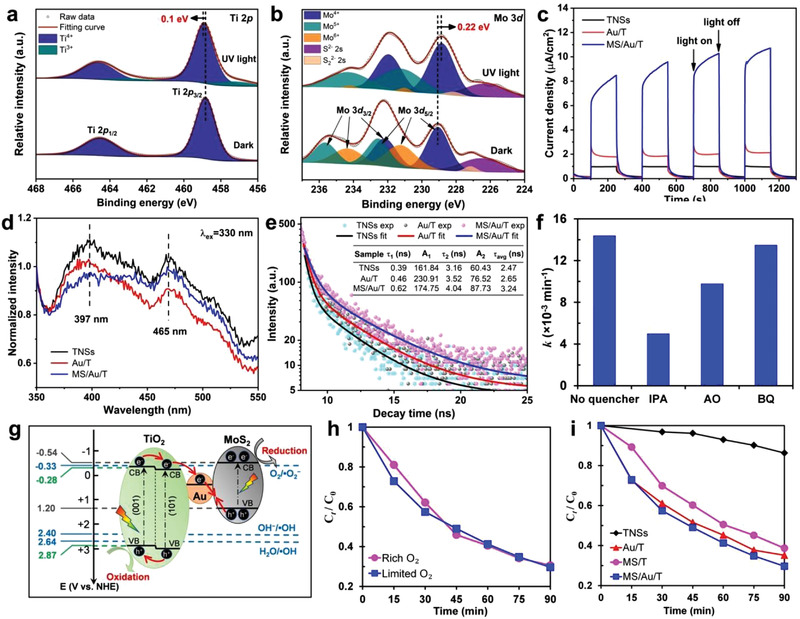
a) ISI‐XPS spectra of Ti 2*p* of 10% MS/Au/T; b) ISI‐XPS spectra of Mo 3*d* of 10% MS/Au/T. c) Photocurrent responses of TNSs, Au/T, and MS/Au/T. d) Normalized steady‐state FL spectra of TNSs, Au/T, and MS/Au/T excited by a wavelength at 330 nm. e) Time‐resolved FL decay of TNSs, Au/T, and MS/Au/T with charge‐carriers' lifetimes inserted accordingly. f) Reactive species experiments for MB photocatalytic degradation. g) Schematic illustration of energy band alignment of TiO_2_, Au, and MoS_2_, and Z‐scheme charge transfer mechanism in MS/Au/T. h) Comparison of the photocatalytic performance of MB degradation with MS/Au/T catalyst under rich and limited O_2_ conditions. i) Comparison of the photocatalytic performance of MB degradation with TNSs, Au/T, 10% MS/T, and 10% MS/Au/T catalysts.

To validate the Z‐scheme charge transfer mechanism, in situ irradiated XPS (ISI‐XPS) analysis of the prepared MS/Au/T photocatalyst were conducted. As shown in **Figure** **4**a, without light irradiation (dark), Ti in MS/Au/T exhibited two peaks at 458.82 eV (Ti 2*p*_3/2_) and 464.52 eV (Ti 2*p*_1/2_), assigned to the characteristics Ti^4+^ species in TiO_2_.^[^ ^30^ ^]^ Under UV light irradiation, a positive shift of 0.1 eV was observed in the BEs of Ti 2*p*, indicating a decrease in electron density.^[^ ^31^ ^]^ Based on the electrostatic shielding effect, more outer electrons resulted in a weaker BE,^[^ ^32^ ^]^ illustrating that the photoinduced electrons were transferred out of TiO_2_. Other two characteristic peaks for Mo^4+^ species in MoS_2_ at 229.04 eV (Mo 3*d*_5/2_) and 232.14 eV (Mo 3*d*_3/2_) were observed in the absence of light,^[^ ^21b^ ^]^ which displayed a negative shift of 0.22 eV upon the light illumination, suggesting the increase of electron density on MoS_2_ surface (Figure 4b). The opposite BE shifts for Ti and Mo elements indicate the pathway for photogenerated electrons transfer across the interfacial heterojunction in MS/Au/T photocatalyst, that is, TiO_2_ → Au → MoS_2_, consistent with Z‐scheme mechanism.

To probe the charge separation behavior, photocurrent transients were performed for TNSs, Au/T, and MS/Au/T, respectively. As indicated in Figure 4c, MS/Au/T displayed the highest photocurrent density, which is eight‐ and four‐fold higher than that of TNSs and Au/T. This reveals the outstanding charge separation capability in our designed system. To further demonstrate the Z‐scheme charge transfer, steady‐state fluorescence (FL) spectra was measured for TNSs, Au/T, and MS/Au/T, respectively. As shown in Figure 4d, two emission peaks at 397 and 465 nm, respectively associated with band‐edge emission and surface‐trapped holes related emission of TiO_2_,^[^ ^33^ ^]^ are both exhibited for all of the three samples. Compared to TiO_2_ and Au/T samples, MS/Au/T has an emission quenching effect at 397 nm but enhanced emission at 465 nm, suggesting a fast trapping or transfer process of free carriers and more surface‐trapped holes in the hetero‐structured catalyst. This is very consistent with the Z‐scheme charge flow, in which the holes on TiO_2_ are preserved but electrons are transferred. Since the photocatalytic reactions are relatively slow processes, the surface‐trapped carriers are more important than free carriers. Time‐resolved FL decay at the emission wavelength of 465 nm was then measured and fitted with a bi‐exponential function^[^ ^34^ ^]^ (Supporting Information) to reveal more dynamics of surface‐trapped holes. As displayed in Figure 4e, MS/Au/T exhibited the slowest decay among the three samples, suggesting the longest lifetime for TiO_2_ surface holes, which was confirmed by the calculated average lifetime *τ* in the inserted table. All of these results demonstrated the Z‐scheme charge transfer mechanism.

To further investigate the photocatalytic mechanism of MS/Au/T, the reactive species in MB degradation under UV light were investigated by using specific reactants. Isopropanol (IPA), ammonium oxalate (AO), and benzoquinone (BQ) were employed as the scavengers for hydroxyl radicals (•OH), holes (h^+^), and superoxide radicals (•O_2_^−^), respectively.^[^ ^35^ ^]^ After introducing IPA, AO, and BQ in MB solution, *k* decreased from 0.0144 min^−1^ to 0.005 min^−1^, 0.098 min^−1^, and 0.0135 min^−1^, respectively, as shown in Figure 4f. This highlighted the critical role of •OH and a modest role of h^+^, as well as the negligible effect of •O_2_^−^ on MB degradation. As one of the major reactive species in photocatalytic reaction, •OH radicals are mainly formed via direct oxidation of OH^−^ or H_2_O by holes or via multiple reduction reactions from •O_2_^−^ by electrons.^[^ ^36^ ^]^ Since •O_2_^−^ had little impact on MB degradation, the oxidation of OH^−^ or H_2_O became the only way to produce •OH radicals. In the synthesized MS/Au/T photocatalyst, the photogenerated holes from the VB of MoS_2_ (1.2 V vs NHE^[^ ^37^ ^]^) are incapable of oxidizing OH^−^ or H_2_O due to their more negative potential than that of OH^−^/•OH (2.4 V vs NHE^[^ ^38^ ^]^) or H_2_O/•OH (2.64 V vs NHE^[^ ^35a^ ^]^), as indicated in Figure 4g. Consequently, •OH radicals must be generated by the photoinduced holes on the VB of TiO_2_ (2.87 V vs NHE, as measured and calculated in Figures S9–S11, Supporting Information).

Based on the charge‐transfer measurements and reactive species experiments, we proposed a photocatalytic mechanism in Figure 4g. The  electrons on VBs of TiO_2_ and MoS_2_ were both excited and transferred to their respective CBs under UV light irradiation. Due to the intimate heterointerface formed between the Au NCs and TNSs, along with the lower Fermi level of Au than that of TiO_2_, the electrons from the CB of TiO_2_ flowed into Au and then recombined with the holes from the VB of MoS_2_. This unique structure preserved the holes with high oxidation ability in VB of TNSs and electrons with high reduction ability in the CB of MoS_2_. As a result, a Z‐scheme charge transfer mechanism was established.^[^ ^10^ ^]^

In many site‐selectively and randomly grown Z‐scheme photocatalytic systems, the photogenerated electrons and holes are separated locally and have many routes to recombine, so a sacrificial agent is usually needed to consume one type of charges (electrons or holes) to guarantee the effective photocatalytic performance of the other type of charges (holes or electrons). In our system, since the holes were proved as the charge carriers to participate in MB degradation, the addition of an electron sink was expected to further improve the photocatalytic activity. Hence, we chose oxygen (O_2_) as an electron sink and performed a comparative test for MB degradation with an MS/Au/T sample in the presence of limited (dissolved O_2_ in solution) and rich O_2_ (under a continuous flow of O_2_ during the test) environment. The results are shown in Figure 4h. It was surprisingly observed that MS/Au/T showed almost the same photodegradation rate under the two conditions, indicating that the excess electrons did not recombine with the holes due to the perfect spatial separation of charges originating from the constructively coupled junctions.

The photocatalytic activity of the optimal MS/Au/T catalyst (10% MS/Au/T) was further compared with pristine TNSs, Au/T, and 10% MS/T by MB degradation experiments. As indicated in Figure 4i, 10% MS/Au‐T showed the highest degradation rate. This is apparently attributed to the synergistic effect among TiO_2_, Au, and MoS_2_, which achieved a high separation efficiency of photoinduced electron‐hole pairs in the designed hetero‐interfacial photocatalyst.

### 2.5 Roles of Electrons in the System

**Figure 5:**
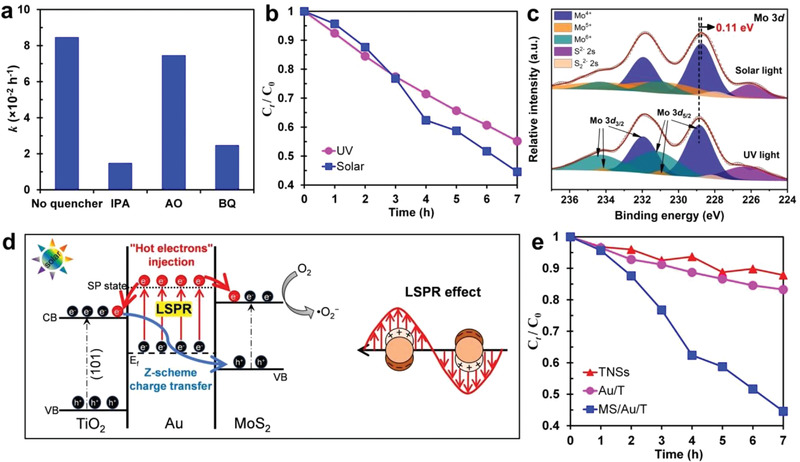
a) Reactive species experiment of 10% MS/Au/T in 4‐NP degradation under UV light; b) Photoactivity of 10% MS/Au/T in 4‐NP degradation under UV and simulated solar light; c) ISI‐XPS spectra of Mo element in 10% MS/Au/T. d) Schematic illustration of “hot electrons” injection‐induced by LSPR of Au under simulated solar light. e) A comparison of photocatalytic activity of TNSs, Au/T, and MS/Au/T for 4‐NP degradation under simulated solar light.

In the designed Z‐scheme heterojunction, the role of holes with high oxidation ability was demonstrated in MB degradation. To investigate the role of electrons in the system, the photocatalytic degradation of 4‐nitrophenol (4‐NP) was performed, because 4‐NP could be oxidized by •OH and also be reduced by •O_2_^−^ into intermediate molecules for further oxidative degradation.^[^ ^39^ ^]^ The reactive species experiments were conducted with the same scavengers as employed in the MB degradation experiment. The corresponding rate constants were calculated and shown in **Figure** **5**a and Figure S12, Supporting Information. After adding AO, *k* slightly decreased, indicating the little effect of holes in the experiment. As IPA and BQ were introduced, *k* dropped significantly, demonstrating that both •OH and •O_2_^−^ were the major reactive species. In general, •O_2_^−^ could reduce pollutants directly or generate •OH via multiple reactions to oxidize the pollutants. Since •OH radicals generated from multiple reactions from •O_2_^−^ was very limited as proven by the above MB degradation, the impact of the BQ test suggested that •O_2_^−^ could directly reduce electron‐deficient compounds bearing nitro groups (4‐NP) into intermediates, which was further oxidized by •OH.^[^ ^39^ ^]^

It is well known that the LSPR of Au could not only broaden the visible light absorption^[^ ^40^ ^]^ but generate “hot electrons” participating in the chemical reactions^[^ ^41^ ^]^ to improve the photocatalytic efficiency. To demonstrate this effect in our system, a comparison test for 4‐NP degradation with 10% MS/Au/T photocatalyst was performed under UV and simulated solar light. As indicated in Figure 5b, within 7 h of irradiation, the degradation rate under simulated solar light exhibited a higher degradation rate (10.6% higher) than that under UV light. The enhanced photocatalytic performance was attributed to “hot electrons” originating from the LSPR of Au. As illustrated in Figure 5d, at a certain wavelength of irradiation, the electrons on the surface of Au NCs were excited and collectively oscillated with the incident electromagnetic field,^[^ ^42^ ^]^ generating “hot electrons” in the surface plasma (SP) state. These “hot electrons” were then transferred to the CBs of the neighboring TiO_2_ and MoS_2_, respectively. The extra electrons on the CB of MoS_2_ reacted with the dissolved O_2_ to generate more •O_2_^−^, promoting the overall degradation rate of 4‐NP.

The described mechanism was proved by comparing the ISI‐XPS patterns of Mo element under UV and solar simulated light, respectively. As shown in Figure 5c, the BE of Mo^4+^ 3*d* decreased by 0.11 eV under simulated solar light compared to the value measured under UV light. This negative shift demonstrated the increase in the electron density, indicating the injection of “hot electrons” from Au NCs to MoS_2_. Hence, Au NCs played dual functions, not only acting as charge mediators in the Z‐scheme structure to boost the interfacial charge recombination but also generating excess “hot electrons” to improve the photocatalytic performance.

In order to demonstrate the superiority of the designed system in the photodegradation of organic pollutants, we compared the photocatalytic degradation of 4‐NP in the presence of TNSs, Au/T, and 10% MS/Au/T photocatalysts under simulated solar light. As indicated in Figure 5e, 10% MS/Au/T exhibited the best photocatalytic activity, followed by Au/T and TNSs. The excellent photoactivity of MS/Au/T was mainly attributed to two factors: i) the coupled junctions in MS/Au/T, which provided a high separation efficiency of photoinduced electron‐hole pairs in the photocatalyst; and ii) the “hot electrons” injection induced by the LSPR effect of Au, which provided extra electrons to participate in the reaction. It was interesting to observe that Au/T only showed a slight improvement in the degradation rate than TNSs, which was opposite to the situation of MB degradation (no LSPR under UV). The possible reason could be as follows: under simulated solar light, there exist two electron‐flow pathways in Au/T. One is from TiO_2_ to Au NCs due to the potential difference between TiO_2_ and Au. The other one is from Au NCs to TiO_2_ due to the “hot electrons” injection. These two routes counteract each other, compromising the charge separation efficiency and leading to only a slight improvement of photocatalytic performance of Au/T than TNSs. This conjecture is evidenced by the nearly unchanged BEs of Ti 2*p* in ISI‐XPS measurements under UV and solar light illumination (Figure S13, Supporting Information). In contrast, MS/Au/T heterostructures could take all the synergetic effects from spatial charge separation and LSPR by Z‐scheme junctions, showing significant performance enhancement in both MB degradation (oxidation only) and 4‐NP degradation (oxidation and reduction).

To further demonstrate the superiority of our photocatalytic system, we summarized the recent research work of 2D/2D Z‐scheme photocatalysts in the photodegradation of dyes and phenols (Table S3, Supporting Information). By comparing the normalized rate constants, our system exhibited notable performance in the degradation of dyes (especially for MB) and comparable performance in the degradation of phenols compared to the other 2D/2D Z‐scheme photocatalysts, which indicates the great potential of our system in the photodegradation of organic pollutants.

## 3 Conclusion

We have designed an efficient MoS_2_/Au/TiO_2_ Z‐scheme photocatalytic system by a novel Au‐induced selective growth of MoS_2_ on the edge sites of 2D TNSs. The ternary structure was fabricated by the following steps: i) hydrothermal preparation of TNSs with highly‐exposed (001) facets; ii) selective deposition of Au NCs on the edge‐sites of TNSs; iii) selective PD of MoS_2_ NFs on Au NCs and (101) facets of TNSs. The edge‐selective growth of MoS_2_ was realized and promoted by Au NCs, which could trap electrons from TNSs and generated excess electrons via the LSPR effect for efficient photoreduction, and offered strong affinity to S for effective MoS_2_ nucleation. The growth and morphological evolution of MoS_2_ could be categorized into three stages: site‐selective nucleation (on Au NCs), facet‐selective extension (along TiO_2_ (101) facets), and random growth (over TiO_2_ (001) facets). It was found that a high precursor concentration and a long PD time promoted random growth. The optimal conditions were found to be a 10% MoS_2_ mass ratio and a 5 h PD time, which could be associated with the morphology of TiO_2_.

ISI‐XPS measurements validated that the photogenerated electrons in TNSs were transferred to Au NCs and recombined with the holes generated in MoS_2_, forming a Z‐scheme charge transfer route. Photocurrent transients and FL measurements further demonstrated the proposed Z‐scheme mechanism. Since this Z‐scheme was built on the TNS (101) facets, a synergetic coupling of the facet junction and the heterojunction was achieved to separate the photogenerated charges in relays. This structure not only suppressed the charge recombination in the system but also maintained the highly exposed surface to the utmost extent. Consequently, the highly exposed surfaces of TNSs and MoS_2_ NFs offered tremendous active sites for redox reactions. The spatial separation of TNSs and MoS_2_ NFs endowed the bifunctional system to participate in different photocatalytic reactions simultaneously. Au NCs in the proposed system served two purposes: constructing the heterojunction and accelerating the redox reactions by supplying “hot electrons”. As a result, the system demonstrated its effectiveness and efficiency in the photodegradation of organic pollutants without involving any sacrificial agents. This work sheds new light on the possibility of designing and selectively fabricating other 2D/2D Z‐scheme photocatalysts using this novel photocatalyst prototype in the future.

## 4 Experimental Section

### Materials

Analytical grade tetrabutyl titanate (C_16_H_36_O_4_Ti, >97%), hydrofluoric acid (HF, 48%), ammonium tetrathiomolybdate (H_8_N_2_MoS_4_, 99.97%), and 4‐nitrophenol (C_6_H_5_NO_3_, ≥99.99%) were procured from Sigma Aldrich. Gold (III) chloride trihydrate (III) (HAuCl_4_·4H_2_O, 99.99%), urea (H_2_NCONH_2_, 99%), and methylene blue (C_16_H_18_ClN_3_S) were purchased from Merck. All the reagents were used as received without further purification. The water used throughout the experiments was purified by a Milli‐Q system.

### Synthesis of TiO_2_ Nanosheets with Highly Exposed (001) Facets

Anatase TNSs were synthesized by hydrothermal method proposed by Han et al.^[^ ^13^ ^]^ In the typical synthesis, 10 mL of tetrabutyl titanate and 1.2 mL of HF solution were mixed in a 25‐mL Teflon stainless‐steel autoclave under ambient conditions, followed by hydrothermal treatment of the solution in a heating oven at 180 °C for 24 h. After cooling down to room temperature, the white precipitate was collected by centrifugation at 6000 rpm, washed with ethanol and deionized (DI) water, and then air dried at 80 °C in an oven for 12 h.

### Synthesis of Edge‐Deposited Au/TiO_2_ Nanosheets

Randomly deposited Au/TNSs were synthesized by an aqueous phase process with urea as a basifier.^[^ ^14b^ ^]^ In the typical synthesis, the as‐prepared anatase TNSs (100 mg) were well dispersed in 10 mL DI water with 60 min in water bath ultra‐sonication followed by 30 min vigorous stirring. Then, 4.2 mm of HAuCl_4_ 4H_2_O was added to the above solution ensuring the random deposition of Au on TNSs. Later, urea was introduced with an amount equivalent to the hundred times than that of the Au precursor to reduce the Au ions. The formed bright‐yellow mixture was thermostatically heated at 80 °C in an oil bath under robust stirring for 3 h. After washing with ethanol and DI water followed by centrifugation several times, the randomly‐deposited Au/TiO_2_ with 4 wt% of Au loading was obtained. After drying the randomly deposited Au/TiO_2_ in a vacuum oven at 90 °C for 3 h, the collected powder was then calcinated from room temperature to 400 °C in an Ar atmosphere under a flow rate of 40 mL min^−1^ with a heating rate of 3 °C min^−1^ for 3 h to obtained the selectively edge‐deposited Au/TiO_2_.

### Synthesis of MoS_2_/Au/TiO_2_ Ternary System

MoS_2_/Au/TiO_2_ photocatalyst was synthesized by a facile one‐step photo‐deposition method.^[^ ^12^ ^]^ In the typical synthesis, 80 mg of as‐synthesized Au/TiO_2_ powder was well dispersed in 30 mL of DI water by 30 min ultrasonication. Meanwhile, a calculated amount of (NH_4_)_2_MoS_4_ was dissolved in 10 mL of DI water, followed by 30 min of vigorous stirring. Then, the prepared (NH_4_)_2_MoS_4_ solution was added into Au/ TiO_2_ suspension dropwise under continuous stirring; subsequently, 4 mL of ethanol was added to the above mixture. The resultant suspension was continuously stirred and bubbled with Ar gas in dark for 30 min to completely remove the dissolved oxygen. Next, the mixture was irradiated by a solar simulator with a 1000 W Xenon short‐arc lamp equipped with an AM1.5G filter for a specific photodeposition time. The sample was then centrifuged and washed with DI water for several times, followed by drying in a heating oven at 70 °C overnight (Figures S14 and S15, Supporting Information). MoS_2_/TiO_2_ samples were synthesized with the same procedures as mentioned above, but using TNSs instead of Au/TiO_2_ as the starting material.

### Characterizations of the Photocatalysts

The crystalline structures of the photocatalysts were characterized by an XRD (Empyrean from PANalytical), using CuK*α* as an X‐ray radiation source (1.5418 Å) and a confocal Raman microscopy spectrometer (WITec alpha300 R) under 532 nm laser light. The detailed morphologies of the samples were studied with a field emission scanning electron microscope (JEOL JSM‐7610F FEG‐SEM) and a TEM (FEI Tecnai TEM 200 kV). The presence and distribution of elements were examined by energy‐dispersive X‐ray spectroscopy (EDS) (results shown in Figures S16 and S17, Supporting Information). HRTEM (FEI Tecnai G2 F20 S‐Twin working at 300 kV) was used to investigate the crystallinity, phase purity, and interfacial lattice. Optical absorptance and reflectance of the prepared samples were measured by UV–vis spectrophotometer (PerkinElmer LAMBDA 1050) to obtain the optical bandgap (results shown in Figures S10 and S11, Supporting Information). The surface chemical states of the synthesized samples were characterized via an XPS equipped with an Al K*α* radiation hemispherical electron energy analyzer (Thermo Fisher ESCALAB 250xi). The BE of C 1s (284.6 eV) was used to calibrate the BE axis. The ISI‐XPS was performed on the same instrument with a 300 W Xenon light (Model: CME‐X305, Microenergy Beijing Technology Co. Ltd., China) equipped with UV and visible light bandpass filter, placed at 10 cm away from the target samples during the analysis.

FL test was conducted using an FS5 spectrometer (Edinburgh Instruments Ltd.). Steady‐state FL spectra were measured under an excitation wavelength at 330 nm. Time‐resolved FL decay was measured with an excitation wavelength at 365 nm and an emission wavelength at 465 nm.

### Photocatalytic Degradation of MB

The photocatalytic MB degradation was carried out in a 200 mL of glass beaker under the irradiation of a flat UV lamp (16.2 W, 360–370 nm). Before irradiation, 20 mg of the prepared photocatalyst was uniformly dispersed in 70 mL of 10 ppm MB aqueous solution kept in dark under magnetic stirring for 30 min to reach an adsorption/desorption equilibrium. The suspension was then irradiated with the UV light under continuously stirring. After given intervals of time, ≈2 mL aliquots were sampled from the reaction beaker with a syringe, centrifuged (10 000 rpm for 8 min), and filtered to remove any residual catalysts. A UV/vis/NIR spectrophotometer (PerkinElmer LAMBDA 1050) was used to measure the absorption spectra of MB aqueous solution as a function of the irradiation time.

### Photocatalytic Degradation of 4‐NP

The photocatalytic degradation of 4‐NP was tested with an irradiation system, which consisted of a tunable solar simulator (1000 W Xenon short arc lamp equipped with AM1.5G Filter), a 400 mL glass beaker with a quartz lid, and an oxygen gas supply. The radiation intensity reaching the surface of the prepared suspension was measured using a radiometer (Delta Ohm 9721) and the matching probe before starting the experiment. Before each set of experiments/cycle, the catalysts were added progressively until the final transmitted radiation intensity reaching the bottom of the beaker went down to 5% of its initial radiation. The pre‐measured specific amount of catalyst was added to 250 mL of 4‐NP suspension with the concentration of 5 mg L^−1^, followed by ultrasonication of 5 min to get the homogenous suspension. Before switching on the lamp, oxygen was bubbled into the suspension for 20 min in dark under magnetic stirring to assure the formation of an adsorption/desorption equilibrium. After that, the solar simulator was turned‐on and oxygen was continuously circulated into the suspension throughout the experiment. After given intervals of time, ≈2 mL aliquots were sampled from the reaction beaker with a syringe, centrifuged (10 000 rpm for 8 min), and filtered to remove any residual catalysts. The filtrate was analyzed with a Thermo Scientific HPLC system (Dionex UltiMate 3000 Photodiode Array Detector).

### Photoelectrochemical Measurement

Photoelectrochemical measurements were carried out on a PGSTAT302N potentiostat (Metrohm Autolab) in a standard three‐electrode cell. A platinum electrode was employed as the counter electrode, and Ag/AgCl electrode was used as the reference electrode. The as‐prepared sample films were coated on fluorine doped tin oxide glass slides as working electrodes (coating area is 1 cm^2^ for the Mott–Schottky experiment, and 1.8 cm^2^ for photocurrent transients). 0.5 m Na_2_SO_4_ solution was filled in the quartz cell as the electrolyte. Mott–Schottky experiment was conducted at a potential step of 50 mV at a constant frequency of 10 kHz. Photocurrent experiment was recorded at a potential of 1 V under the irradiation of a 50 W LED lamp with a wavelength of 365 nm.

## Conflict of Interest

The authors declare no conflict of interest.

## Supporting information

Supporting Information

## Data Availability

The data that support the findings of this study are available in the supplementary material of this article.
