# Supplementary material for: Coupling Long‐Range Facet Junction and Interfacial Heterojunction via Edge‐Selective Deposition for High‐Performance Z‐Scheme Photocatalyst
Source: Adv Sci (Weinh). 2022 Apr 24;9(18):2200346. doi: 10.1002/advs.202200346 (PMC9218749; doi:10.1002/advs.202200346)
Supplement: Supplementary file 1 — Supporting Information [file ADVS-9-2200346-s001.pdf]

ADVANCED SCIENCE
Open Access

Supporting Information

for *Adv. Sci.*, DOI 10.1002/advs.202200346

Coupling Long-Range Facet Junction and Interfacial Heterojunction via Edge-Selective Deposition for High-Performance Z-Scheme Photocatalyst

*Xuan Li, Shoaib Anwer, Qiangshun Guan, Dalaver H. Anjum, Giovanni Palmisano and Lianxi Zheng**

Supporting Information

Coupling Long-Range Facet Junction and Interfacial Heterojunction *via* Edge-Selective Deposition for High-Performance Z-Scheme Photocatalyst

*Xuan Li, Shoaib Anwer, Qiangshun Guan, Dalaver H. Anjum, Giovanni Palmisano, and Lianxi Zheng**

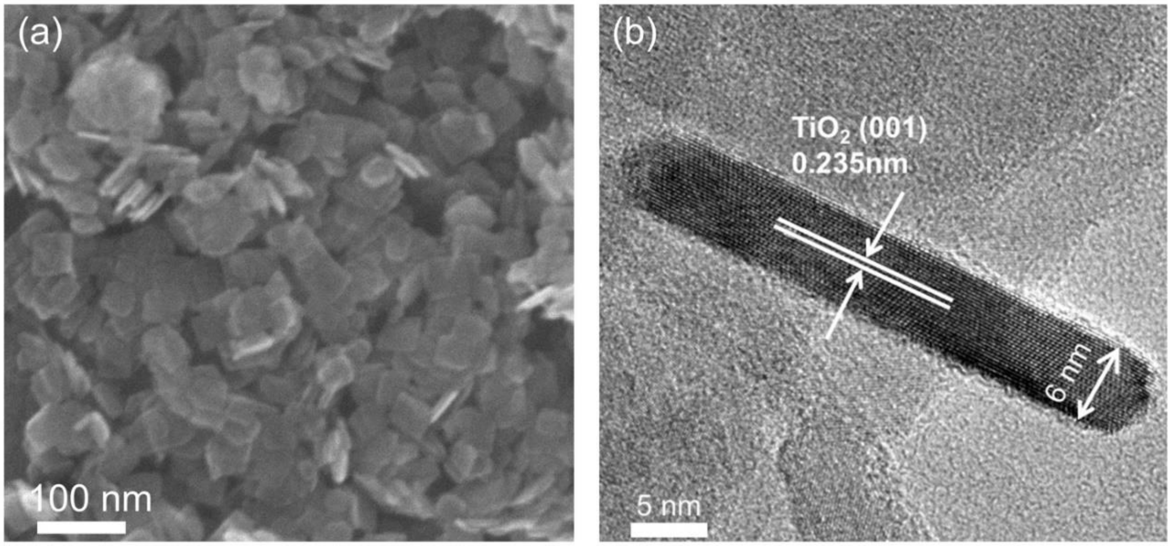

Figure S1. (a) SEM image of TiO_2 to show the nanosheet morphology; (b) HRTEM image from the vertical side of TiO_2 nanosheets.

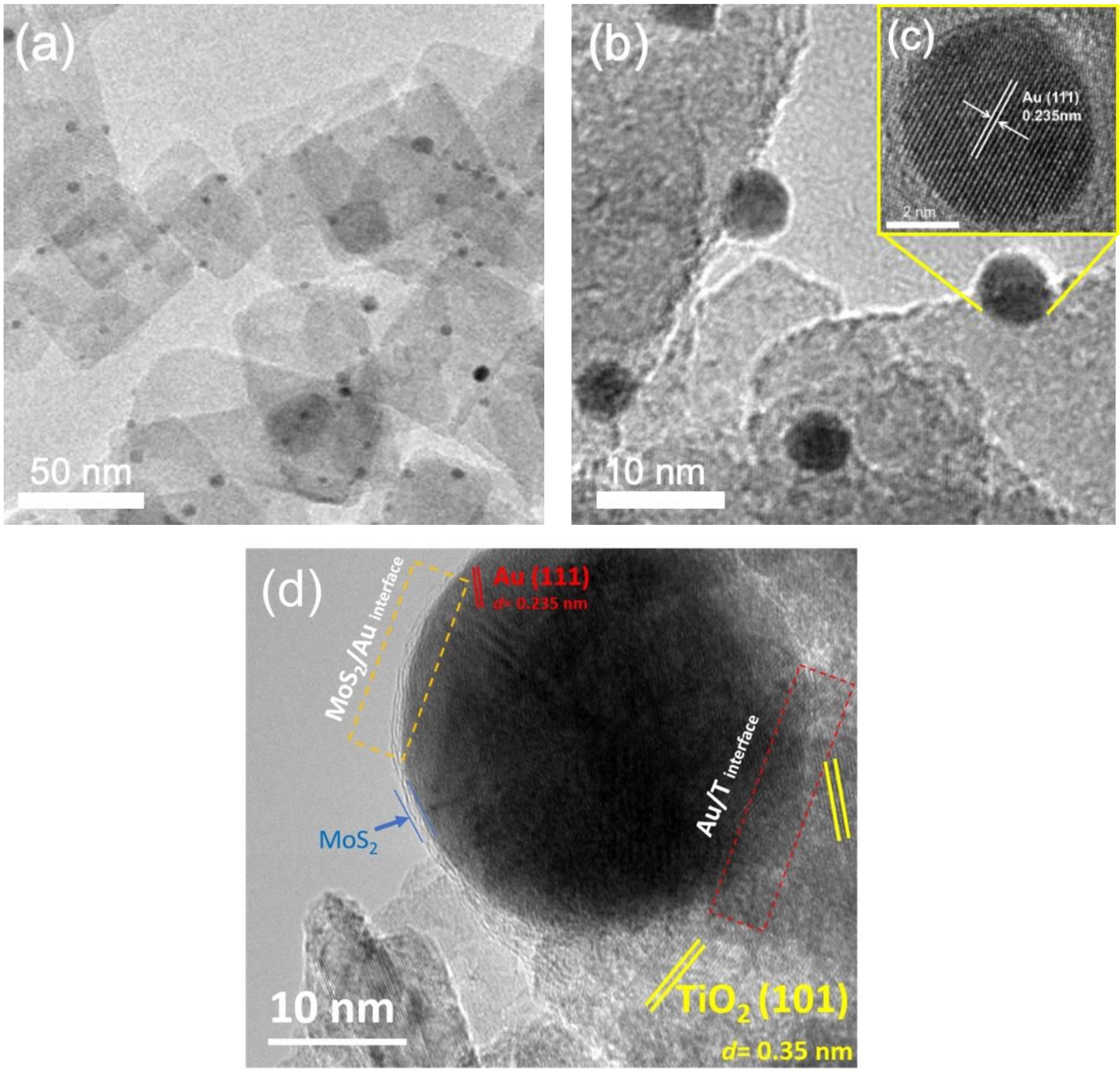

Figure S2. (a) Large-scale TEM image of edge-deposited Au/TiO_2; (b) HRTEM image of edge-deposited Au/TiO_2 showing the close contact between Au NCs and anatase TNSs; (c) Lattice spacing of (111) facet of Au NCs; (d) HRTEM image showing two interfaces of TiO_2/Au and Au/MoS_2 in MS/Au/T heterostructure.

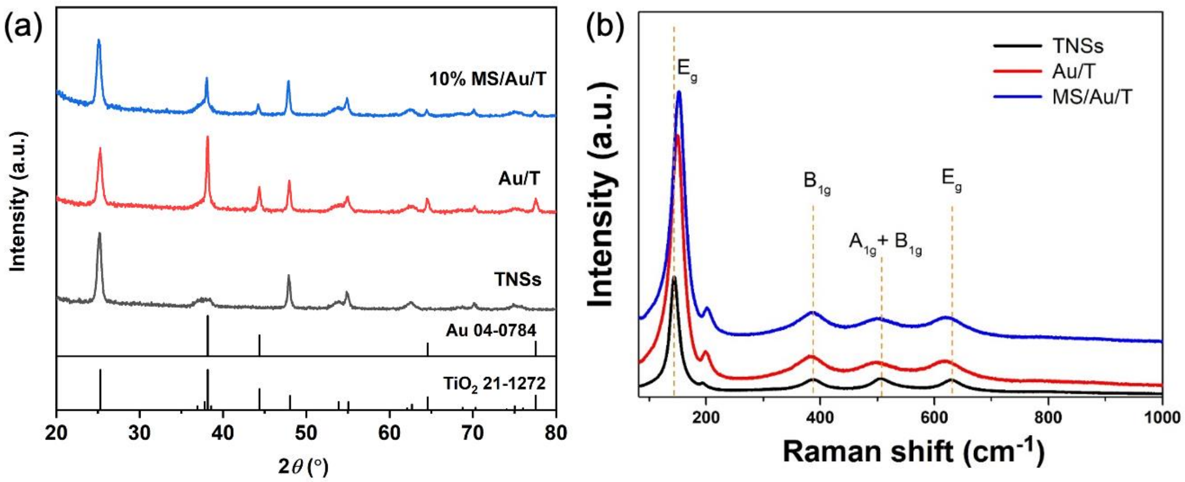

Figure S3. (a) XRD patterns and (b) Raman spectra of the prepared anatase TNSs, Au/T, and 10% MS/Au/T.

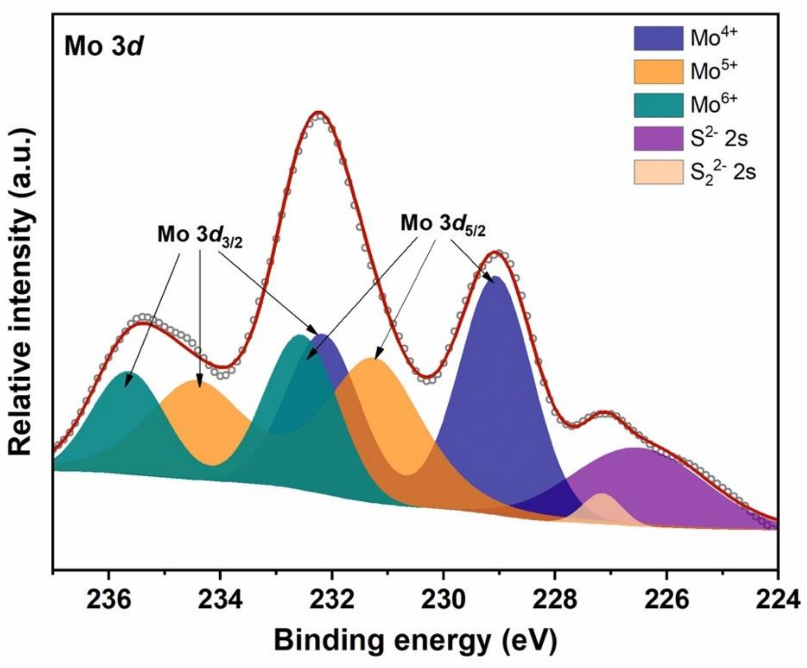

Figure S4. XPS spectra of Mo 3*d* in 10% MS/Au/T.

Table S1. XPS fitting results for Mo and S elements in 10% MS/Au/T in the dark, under UV and UV-vis light, respectively.

Conditions	Element	Orbital	BE position [eV]	FWHM [eV]	Area
**Dark**	Mo^{4+}	$3d_{5/2}$	229.04	1.56	7307.08
		$3d_{3/2}$	232.14		4871.39
	Mo^{5+}	$3d_{5/2}$	231.22	2.04	5019.84
		$3d_{3/2}$	234.32		3346.56
	Mo^{6+}	$3d_{5/2}$	232.53	1.55	4922.51
		$3d_{3/2}$	235.63		3281.67
	S^{2-}	$2s$	226.50	2.80	3934.13
	S_2^{2-}	$2s$	227.14	0.82	462.65
	S^{2-}	$2p_{3/2}$	161.88	1.89	4970.49
		$2p_{1/2}$	163.08		2485.24
	S_2^{2-}	$2p_{3/2}$	163.10	1.33	3611.30
		$2p_{1/2}$	164.30		1805.65
**UV**	Mo^{4+}	$3d_{5/2}$	228.82	1.75	8720.57
		$3d_{3/2}$	231.92		5813.72
	Mo^{5+}	$3d_{5/2}$	230.99	0.69	271.60
		$3d_{3/2}$	234.17		181.07
	Mo^{6+}	$3d_{5/2}$	231.18	2.83	6128.0
		$3d_{3/2}$	234.28		4085.34
	S^{2-}	$2s$	226.33	2.77	3567.51
	S_2^{2-}	$2s$	228.10	1.10	423.60
	S^{2-}	$2p_{3/2}$	161.55	1.43	4479.49
		$2p_{1/2}$	162.75		2239.75
	S_2^{2-}	$2p_{3/2}$	162.79	1.39	2945.26
		$2p_{1/2}$	163.99		1472.63
**UV-vis/Solar**	Mo^{4+}	$3d_{5/2}$	228.71	1.74	8133.44
		$3d_{3/2}$	231.91		5422.30
	Mo^{5+}	$3d_{5/2}$	230.86	4.91	5352.40
		$3d_{3/2}$	234.06		3568.27
	Mo^{6+}	$3d_{5/2}$	231.06	2.16	2412.63
		$3d_{3/2}$	234.26		1608.42
	S^{2-}	$2s$	226.05	1.91	2407.69
	S_2^{2-}	$2s$	227.91	1.04	787.95
	S^{2-}	$2p_{3/2}$	161.44	1.36	4207.91
		$2p_{1/2}$	162.64		2103.96
	S_2^{2-}	$2p_{3/2}$	162.82	1.67	3342.83
		$2p_{1/2}$	164.02		1671.42

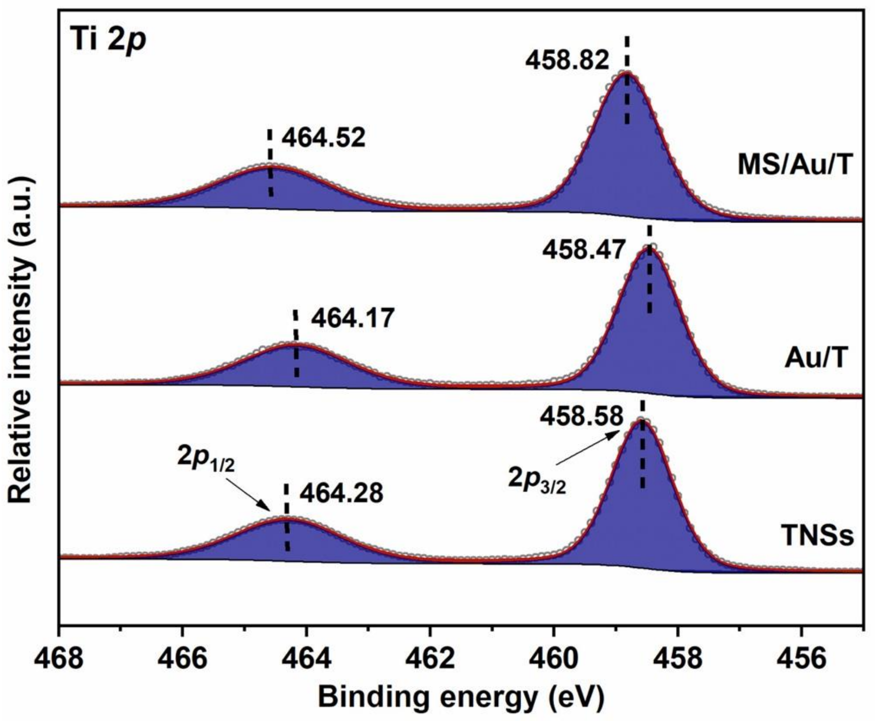

Figure S5. XPS spectra of Ti 2*p* in TNSs, Au/T and 10% MS/Au/T.

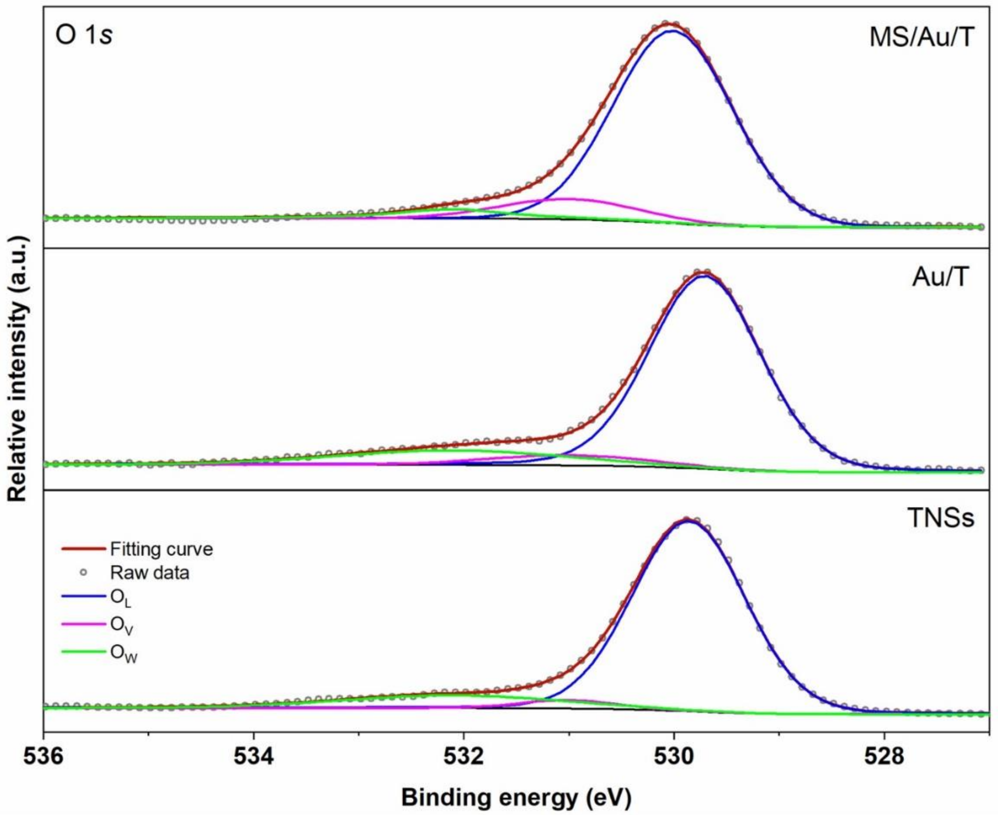

Figure S6. XPS spectra of O 1*s* in TNSs, Au/T and 10% MS/Au/T.

Table S2. XPS fitting results for O elements in TNSs, Au/T, and 10% MS/Au/T.

Sample	Element	BE [eV]	FWHM [eV]	Area	Percentage
TNSs	O_L	529.9	1.26	145374.10	85.7%
	O_V	531.0	1.06	5887.56	3.5%
	O_W	532.1	2.47	18428.23	10.9%
Au/T	O_L	529.7	1.25	169952.40	81.9%
	O_V	531.0	1.81	12463.70	6.0%
	O_W	532.1	2.60	25039.25	12.1%
MS/Au/T	O_L	530.0	1.33	161169.90	84.6%
	O_V	531.0	1.53	19183.17	10.1%
	O_W	532.1	1.28	10149.54	5.3%

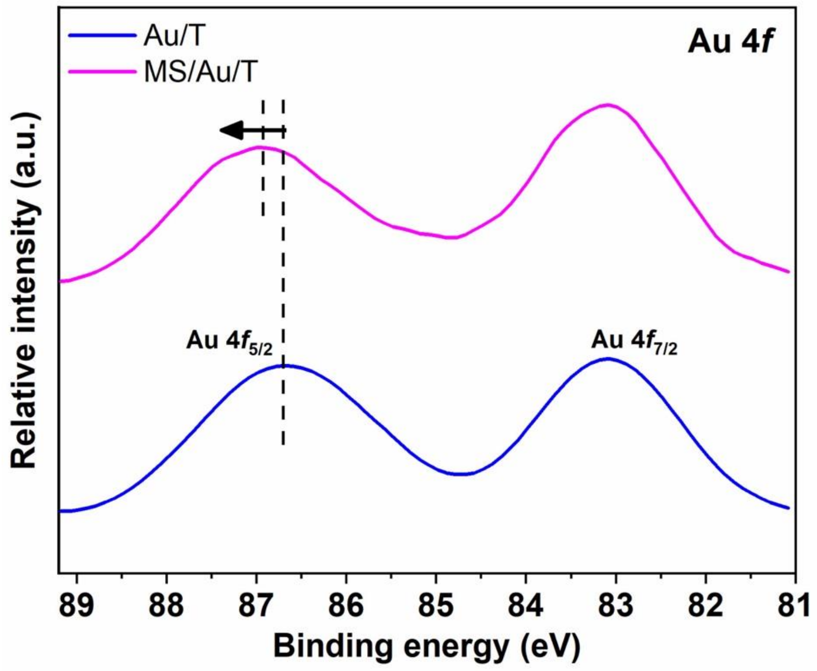

Figure S7. XPS spectra of Au 4*f* in Au/T and 10% MS/Au/T.

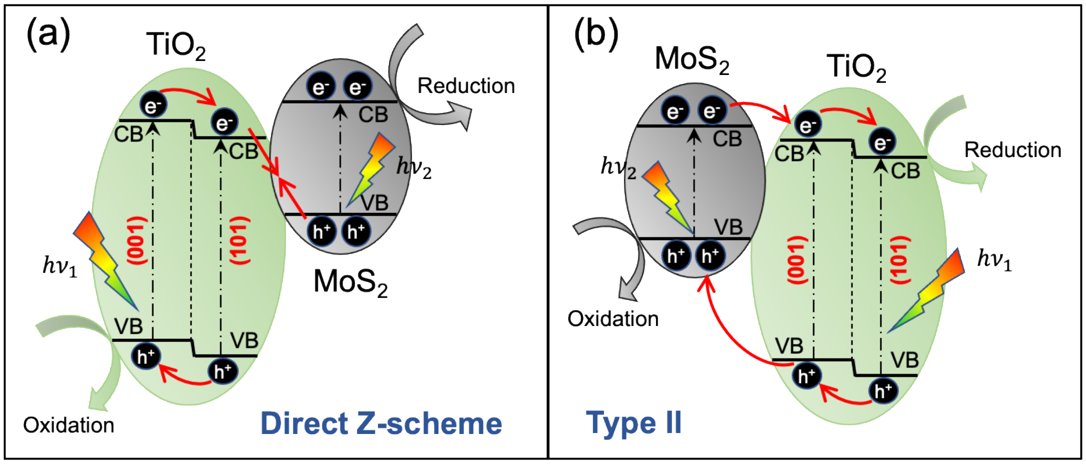

Figure S8. Schematic of charge transfer in (a) direct Z-scheme and (b) Type II heterostructure, respectively.

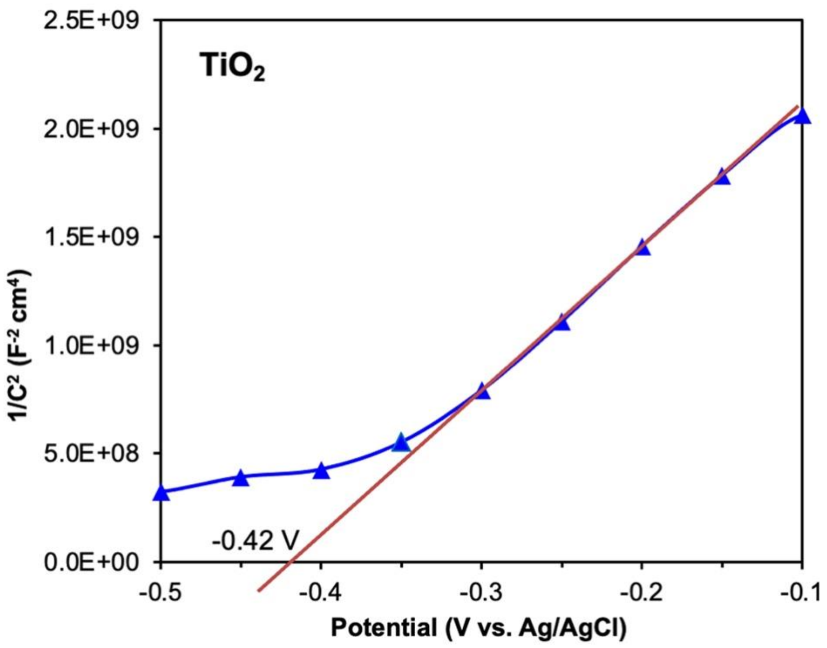

Figure S9. Mott-Schottky plot for determination of flat band potential of TiO_2.

The measured flat band potential relative to Ag/AgCl reference electrode can be converted to the reversible hydrogen electrode (RHE) according to the Nernst equation:[1]

$$E_{RHE} = E_{Ag/AgCl} + E^{\circ}_{Ag/AgCl} + 0.059pH$$

where E_{RHE} is the converted potential vs. RHE, $E_{Ag/AgCl}$ is the measured potential against Ag/AgCl reference electrode, $E^{\circ}_{Ag/AgCl}$ is the standard potential of Ag/AgCl at 25 °C (0.1976 V), and the pH value of the electrolyte is measured around 6.8.

According to the above equation, the calculated flat band potential (E_{RHE}) is -0.18 V. As the flat band potential is about 0.1-0.3 V below the CB minimum (CBM) for undoped n-type semiconductor, the CBM of TiO_2 is determined approximately to be -0.28 V. According to the absorptance and Tauc plot (Figure S10-S11), the band gap of pristine TiO_2 is 3.15 eV. The VBM is thus calculated to be 2.87 V.

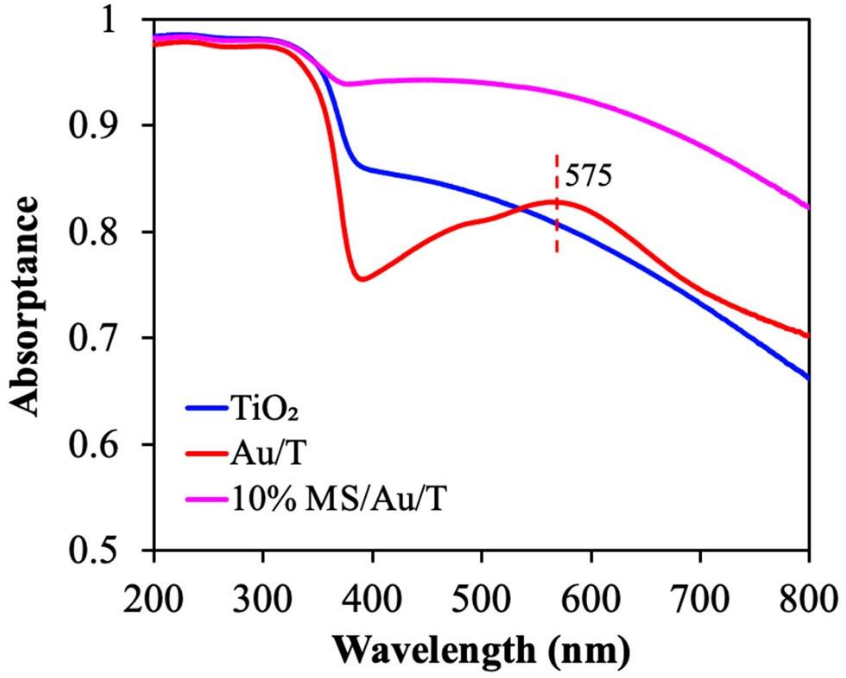

Figure S10. UV–vis absorption spectra of pristine TiO_2, Au/T (with indication of LSPR peak), and 10% MS/Au/T.

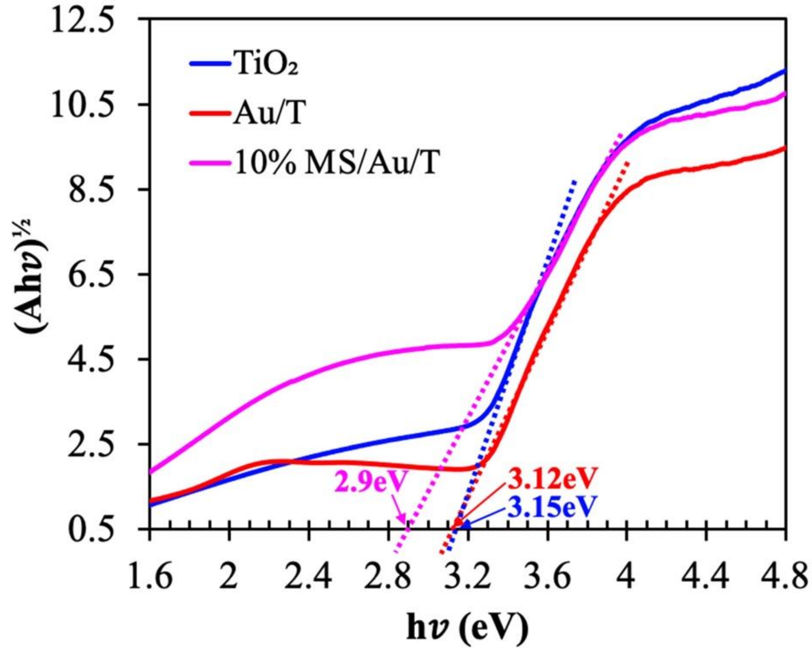

Figure S11. Tauc-plot analysis indicating the band gap of pristine TiO_2, Au/T, and 10% MS/Au/T.

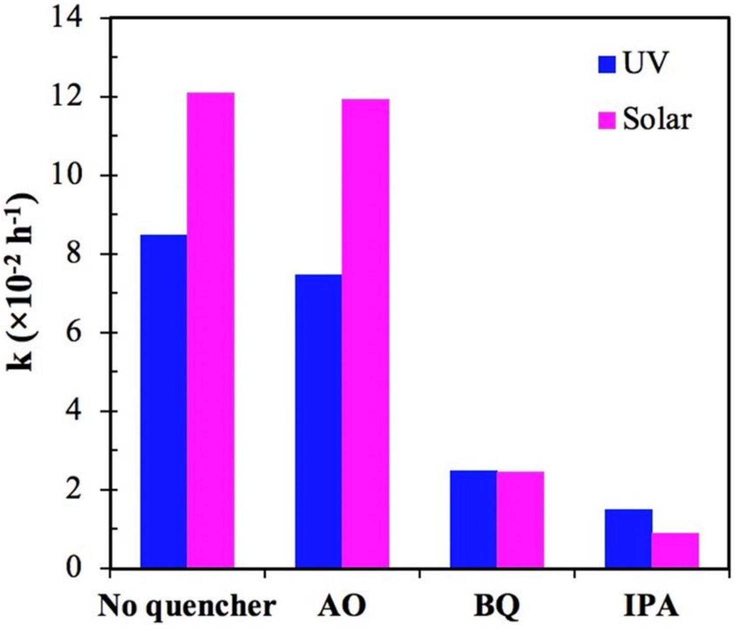

Figure S12. Reactive species experiment of 4-NP degradation with 10% MS/Au/T under UV and simulated solar light, respectively.

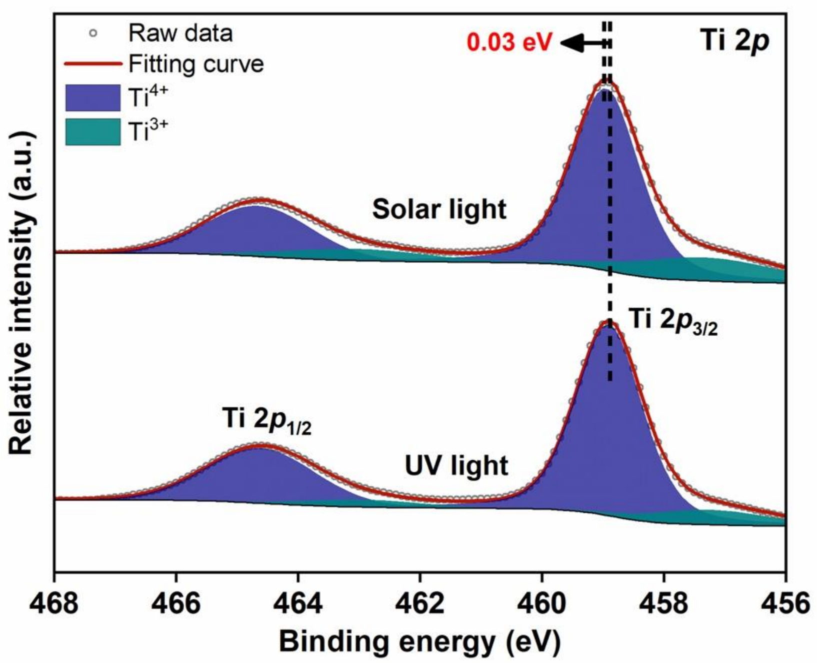

Figure S13. ISI-XPS spectra of Ti 2p of 10% MS/Au/T under UV and simulated solar light.

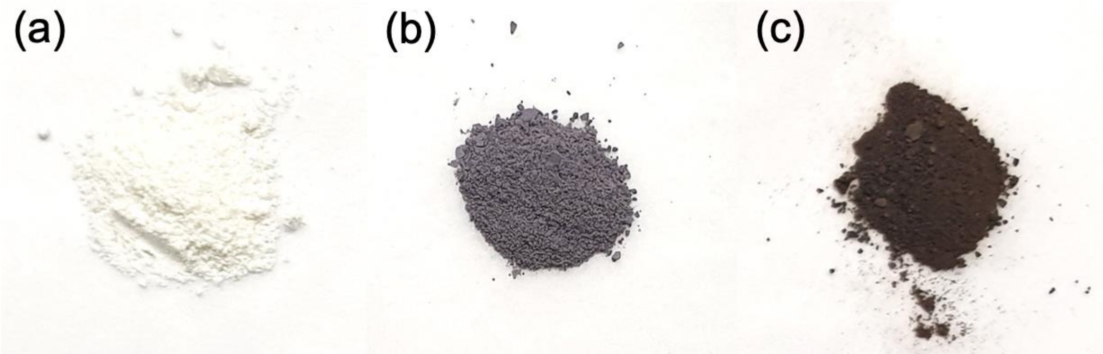

Figure S14. Color changing of the as-synthesized samples: (a) TNSs, (b) Au/T and (c) MS/Au/T.

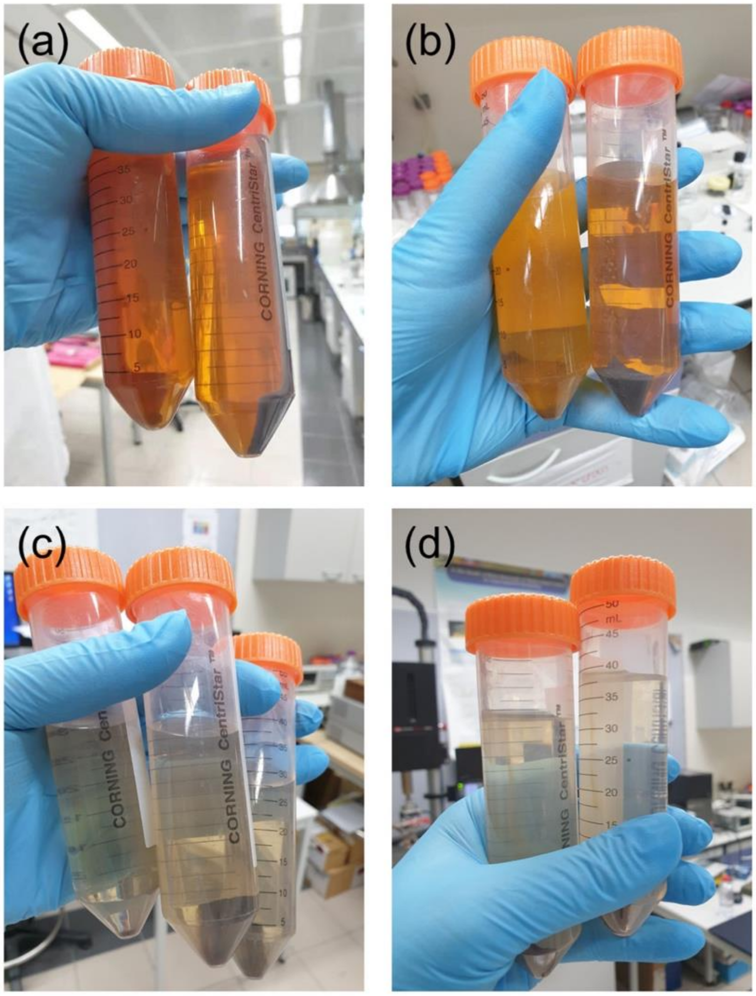

Figure S15. Color changing of the residual solution of 10% MS/Au/T nanocomposite by the first centrifugation after different PD time: (a) 1h, (b) 2h, (c) 4h and (d) 6h.

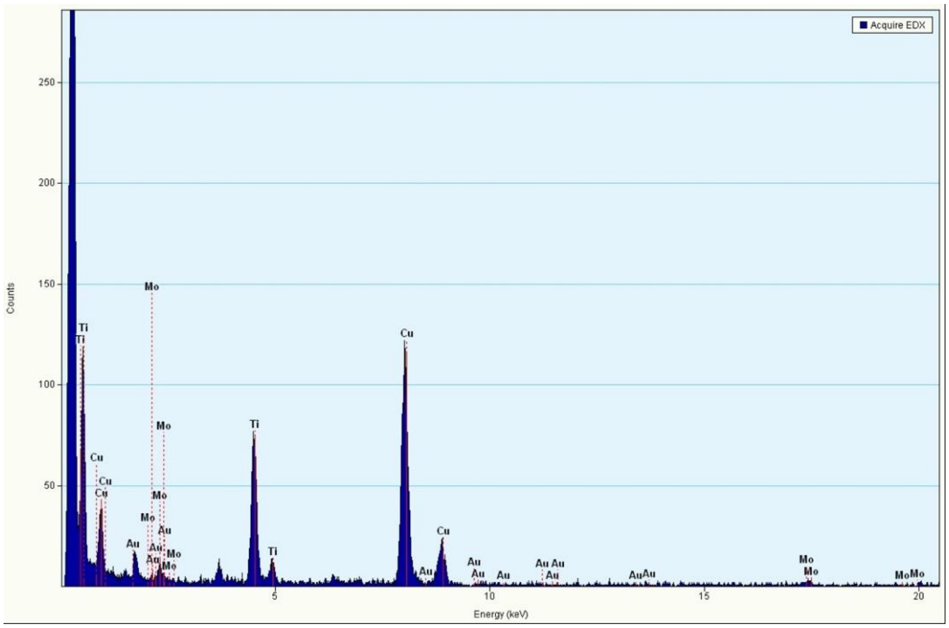

Figure S16. EDS spectra of Ti, Au and Mo elements in MS/Au/T.

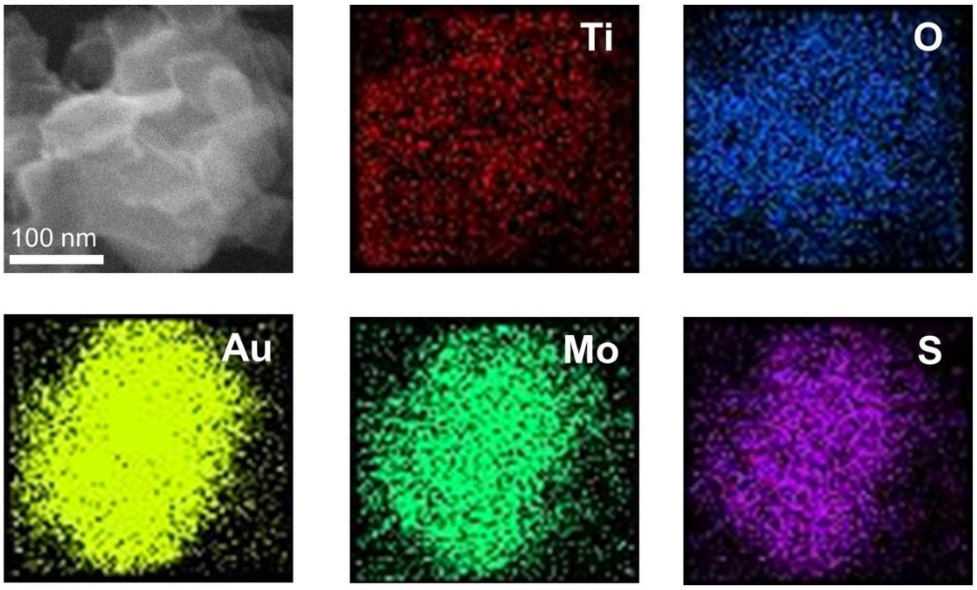

Figure S17. SEM-EDX mapping of Ti, O, Au, Mo and S in MS/Au/T.

Table S3. Recent research work of 2D/2D Z-scheme photocatalysts in the photodegradation of dyes and phenols.

2D/2D Z-scheme photocatalyst [a]	Light source	Pollutant [b] (mg/L)	C_{cat}/C_{pollut} [c]	Removal efficiency	Rate constant *k* ($\times 10^{-2}$/min)	Normalized rate constant *k* [d] ($\times 10^{-4}$/min)	Ref
$SnNb_2O_6$/ZnO	300 W Xe	RhB (10)	50	95% in 60 min	5.26	10.52	[2]
Bi_3O_4Cl/gCN	250 W Xe	RhB (10)	50	98.3% in 90 min	4.53	9.06	[3]
gCN/MnO_2	300 W Xe	RhB (10)	100	91.3% in 60 min	3.3	3.3	[4]
Bi_2WO_6/gCN	500 W tungsten	RhB (10)	50	98% in 60 min	4.3	8.6	[5]
$WO_3/SnNb_2O_6$	500 W tungsten	RhB (10)	100	93.4% in 180 min	1.5	1.5	[6]
PVDF@gCN/BWNO	500 W Xe	RhB (10)	100	100% in 150 min	2.01	2.01	[7]
gCN/TiO_2	300 W Xe	MO (10)	100	90% in 90 min	4.71	4.71	[8]
$Ti_3C_2T_x$@$BiVO_4$/CdS	200 W Hg	MB (6.4)	156.3	85% in 60 min	3.16	2.02	[9]
Bi_2MoO_6/gCN	–	MB (20)	10	92.7% in 150 min	0.23	2.3	[10]
MoS_2/Au/TiO_2	16.2 W LED	MB (10)	28.6	88.1% in 90 min	1.44	5.03	this work
gCN/$Bi_{20}TiO_{32}$	300 W Xe	4-CP (12.9)	77.76	100% in 60 min	6.67	8.57	[10]
C@WS_2/gCN	300 W Xe	2,4-DCP (10)	100	41.3% in 180 min	1.04	1.04	[11]
$SnNb_2O_6$/ZnO	300 W Xe	phenol (20)	50	96% in 120 min	2.6	5.2	[2]
gCN/MnO_2	300 W Xe	phenol (50)	20	73.6% in 3 h	0.74	3.7	[4]
BiOBr/UMOFNs	300 W Xe	phenol (10)	66.7	99% in 270 min	1.28	1.92	[12]
BiOCl/gCN	500 W Xe	phenol (40)	25	94.3% in 105 min	0.22	0.8	[13]
MoS_2/Au/TiO_2	16.2 W LED	4-NP (5)	8	55.4% in 7 h	0.2	2.5	this work

Note: [a]gCN, g-C_3N_4; BWNO, $Bi_2W_{0.98}Nb_{0.02}O_6$; UMOFNs, ultrathin MOF nanosheets.
[b]RhB, rhodamine B; MO, methyl orange; 4-CP, 4-chlorophenol; 2,4-DCP, 2,4-dichlorophenol.
[c]C_{cat}/C_{pollut}, concentration ratio of catalyst/pollutant;
[d] normalized *k*, rate constants normalized to the concentration ratio of catalyst/pollutant.

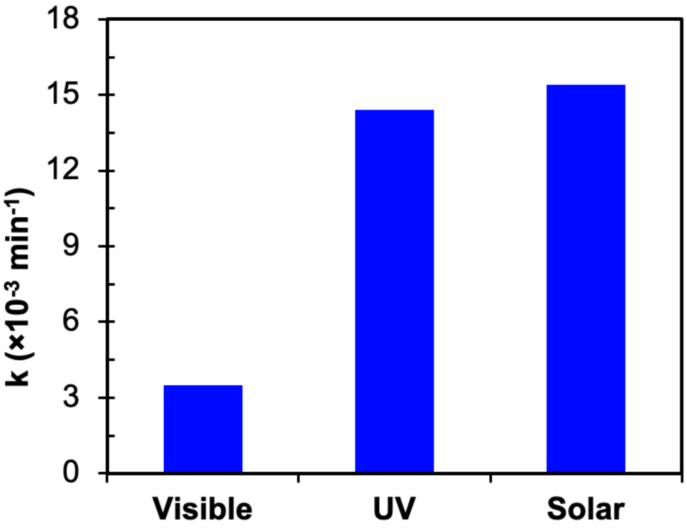

Figure S18. Photocatalytic performance of MB degradation with 10% MS/Au/T under visible, UV and simulated solar light, respectively.

Calculation of pseudo-first order constant (*k*)

The photodegradation of 4-NP is modeled *via* an exponential fitting, from which the pseudo-first order constant (*k*) can be derived according to the following equations:[14]

$$-r_{4-NP} = -\frac{V}{X}\frac{dC_{4-NP}}{dt} = kC_{4-NP}$$

where the disappearance rate of 4-NP ($-r_{4-NP}$) is a function of the concentration of 4-NP (C_{4-NP}) multiplied by a pseudo-first order constant (*k*). V is the volume of the reactor; X represents either the mass or the surface area of the photocatalyst in the reactor; and t is the irradiation time.

Curve fitting of time-resolved FL decay

A bi-exponential function is employed to fit the FL decay curves mathematically.[15]

$$y = y_0 + A_1 \exp(-x/\tau_1) + A_2 \exp(-x/\tau_2)$$

where y_0 is the basal constant; A_1 and A_2 are the amplitude coefficient; τ_1 and τ_2 are the fluorescent lifetimes corresponding to the trapping process and recombination process,[16] respectively.

The average fluorescent lifetime can be calculated by the following equation.[15]

$$\tau = \frac{A_1\tau_1^2 + A_2\tau_2^2}{A_1\tau_1 + A_2\tau_2}$$

References

[1] W. Yin, L. Bai, Y. Zhu, S. Zhong, L. Zhao, Z. Li, S. Bai, *ACS Appl. Mater. Interfaces* **2016**, 8, 23133.

[2] H. Wang, J. Yu, X. Zhan, L. Chen, Y. Sun, H. Shi, *Appl. Surf. Sci.* **2020**, 528, 146938.

[3] H. Che, G. Che, H. Dong, W. Hu, H. Hu, C. Liu, C. Li, *Appl. Surf. Sci.* **2018**, 455, 705.

[4] P. Xia, B. Zhu, B. Cheng, J. Yu, J. Xu, *ACS Sustain. Chem. Eng.* **2018**, 6, 965.

[5] W. Guo, K. Fan, J. Zhang, C. Xu, *Appl. Surf. Sci.* **2018**, 447, 125.

[6] X. Ma, W. Ma, D. Jiang, D. Li, S. Meng, M. Chen, *J. Colloid Interface Sci.* **2017**, 506, 93.

[7] C. Wu, H. Yan, J. Zhong, J. Xie, D. Wang, Y. Shi, L. Zhang, J. Zhu, Q. Chen, *Appl. Surf. Sci.* **2019**, 496, 143731.

[8] B. Chen, P. Li, S. Zhang, W. Zhang, X. Dong, F. Xi, J. Liu, *J. Colloid Interface Sci.* **2016**, 478, 263.

[9] W. Wang, Z. D. Hood, X. Zhang, I. N. Ivanov, Z. Bao, T. Su, M. Jin, L. Bai, X. Wang, R. Zhang, Z. Wu, *ChemCatChem* **2020**, 12, 3334.

[10] R. Guo, X. Zhang, B. Li, H. Zhang, J. Gou, X. Cheng, *J. Phys. D: Appl. Phys.* **2018**, 52, 085302.

[11] S. Wu, Y. Wang, *Chemosphere* **2021**, 273, 129746.

[12] B. Lin, Z. Chen, L. Shui, G. Zhou, X. Wang, *Nanotechnology* **2020**, 32, 045711.

[13] H. Ma, J. Liu, S. Zuo, Y. Yu, W. Liu, Y. Wang, B. Li, *ChemistrySelect* **2021**, 6, 10097.

[14] L. Y. Ozer, H. Apostoleris, F. Ravaux, S. I. Shylin, F. Mamedov, A. Lindblad, F. O. Johansson, M. Chiesa, J. Sá, G. Palmisano, *ChemCatChem* **2018**, 10, 2949.

[15] L. Guo, Z. Yang, K. Marcus, Z. Li, B. Luo, L. Zhou, X. Wang, Y. Du, Y. Yang, *Energy Environ. Sci.* **2018**, 11, 106.

[16] Z. Yan, W. Wang, L. Du, J. Zhu, D. L. Phillips, J. Xu, *Appl. Catal., B* **2020**, 275, 119151.
